# A Mechanistic Model for Predicting Cell Surface Presentation of Competing Peptides by MHC Class I Molecules

**DOI:** 10.3389/fimmu.2018.01538

**Published:** 2018-07-05

**Authors:** Denise S. M. Boulanger, Ruth C. Eccleston, Andrew Phillips, Peter V. Coveney, Tim Elliott, Neil Dalchau

**Affiliations:** ^1^Centre for Cancer Immunology and Institute for Life Sciences, Faculty of Medicine, University of Southampton, Southampton, United Kingdom; ^2^Centre for Computational Science, Department of Chemistry, University College London, London, United Kingdom; ^3^CoMPLEX, University College London, London, United Kingdom; ^4^Microsoft Research, Cambridge, United Kingdom

**Keywords:** antigen presentation, major histocompatibility class I, mechanistic model, interferon-γ, peptide competition, abundance

## Abstract

Major histocompatibility complex-I (MHC-I) molecules play a central role in the immune response to viruses and cancers. They present peptides on the surface of affected cells, for recognition by cytotoxic T cells. Determining which peptides are presented, and in what proportion, has profound implications for developing effective, medical treatments. However, our ability to predict peptide presentation levels is currently limited. Existing prediction algorithms focus primarily on the binding affinity of peptides to MHC-I, and do not predict the relative abundance of individual peptides on the surface of antigen-presenting cells *in situ* which is a critical parameter for determining the strength and specificity of the ensuing immune response. Here, we develop and experimentally verify a mechanistic model for predicting cell-surface presentation of competing peptides. Our approach explicitly models key steps in the processing of intracellular peptides, incorporating both peptide binding affinity and intracellular peptide abundance. We use the resulting model to predict how the peptide repertoire is modified by interferon-γ, an immune modulator well known to enhance expression of antigen processing and presentation proteins.

## Introduction

Cellular immunity has a major role in resistance to infection and cancer. CD8 T cells play an important part, by recognizing protein fragments (peptides) that are generated within an infected or cancerous cell and presented on the cell surface by class I major histocompatibility complex (MHC-I) molecules. The recognition of a specific peptide bound to MHC-I, called a peptide–MHC complex (pMHC), is achieved by the T cell receptor (TCR). The abundance of infection- or cancer-specific pMHC complexes on the cell surface is a key factor in the development of an efficient T cell response, where high abundance has been associated with an immunodominance phenomenon in which immune responses focus on only a few of the many potential peptides (1, 2). Other factors influencing the efficiency of the T cell response include the frequency of T cell precursors of a certain specificity (3), and the affinity of the TCR to its multiple target pMHC complexes. In viral infections, additional factors such as the temporal appearance of peptides that trigger an immune response (epitopes) also have a major impact (4).

Development of efficient vaccines or immunotherapies relies on the identification of peptides that can be presented in high abundance on the cell surface. In the field of cancer immunotherapy, recent development of high-throughput gene sequencing has generated catalogs of mutations found in individual tumors (mutanomes) and has led to the identification of large numbers of novel peptides, generated as a result of a mutation and only present in some tumor cells (neo-epitopes), that can potentially be targeted for vaccine development or immunotherapy (5, 6). T cell immunotherapy has gained widespread interest following successful treatments in the clinic [reviewed in Pardoll (7, 8)], and could be further enhanced with anti-cancer vaccines that elicit strong CD8 T cell responses to tumor-specific peptides. Success of those strategies will rely on the choice of the most appropriate peptides, and good predictive methods could narrow down the number of candidate peptides to a realistically testable number.

The abundance of a given pMHC complex on the cell surface is determined partly by the affinity of the peptide to the MHC-I molecule, and is further controlled by the antigen processing and presentation machinery. Peptides binding to MHC-I molecules are produced by proteasomal degradation of newly synthesized defective ribosomal products, short-lived proteins, or of fully mature proteins naturally degrading over time (retirees) (9–12). Peptides enter the endoplasmic reticulum (ER) *via* the transporter associated with antigen processing (TAP) and compete for binding to an MHC-I molecule within the peptide loading complex, comprising TAP and the chaperone molecules, such as tapasin, calreticulin, and ERp57 [reviewed in Van Hateren et al. (13)]. The absence of each of these chaperones affects the overall cell surface abundance of peptide, but the absence of tapasin has the additional effect of modifying the relative proportions of these peptides (14–16) and consequently the CD8 T cell immunodominance hierarchy (17, 18).

The affinity of a peptide for a specific MHC-I molecule can be directly measured experimentally, which has aided the development of algorithms predicting the affinity of any peptide for specific MHC-I alleles based on the sequence of the peptide [BIMAS (19, 20), NetMHC (21)]. These algorithms have been improved over time and can also include proteasomal cleavage and TAP transport predictions [IEDB (22)]. However, the identification of cell surface peptide repertoires, made possible by the development of high-throughput mass spectrometry technology (23, 24) showed in several cases that predicted peptide affinity to MHC-I has poor correlation with cell surface abundance [(25) Supplementary Figure]. We propose, therefore, that improving the prediction of cell surface abundance of pMHC complexes requires peptide sequence-based algorithms to be combined with known mechanisms of the antigen processing and presentation pathway (26). These mechanisms include the phenomenon of cofactor-assisted loading of peptides onto MHC-I by tapasin, the rate of generation of peptides and their intracellular abundance. These may be linked to the abundance of the source proteins (25, 27) and their degradation rates (27, 28), as well as to the rate of translation of the source proteins (29). Poor correlations between cell surface abundance of pMHC and each of these factors individually have been observed [source protein abundance (25, 30) and peptide affinity (25)]. We hypothesize that these factors need to be appropriately incorporated within a mechanistic model in order to obtain good predictions.

We have previously developed mathematical models that simulate cell surface abundance of multiple peptides bound to MHC-I, at steady-state on the surface of living cells, and incorporate variations in peptide supply and peptide affinity to MHC-I (31, 32). In this context, a high affinity peptide is defined as having a low off-rate, unbinding slowly from MHC-I. The models were used to interpret how tapasin could preferentially select peptides that form stable complexes with MHC-I molecules, and further suggest how MHC haplotypes differ in the extent of their tapasin-mediated selection, some haplotypes have the intrinsic ability to select and assemble with optimal peptides independent of tapasin whereas others are dependent on tapasin to be stably loaded. A key quantitative prediction of the models was that equilibrium cell surface abundance of a given peptide (*P_i_*) bound to MHC at the cell surface (*Me*) can be approximated by the following filtering relation:

(1) $$\left[MeP_{i}\right]\approx \frac{g_{i}}{u_{i}{\;}^{\alpha }}$$

where *g_i_* is the supply of the peptide *via* TAP and *u_i_* is the rate of dissociation of the peptide from MHC-I. We found that the exponent α is increased by tapasin, leading to greater filtering of peptides based on their off-rate from MHC. The model has also been used to simulate the competition of peptides for cell surface presentation (32). However, predictions for the direct competition between peptides of known supply and affinity to MHC have so far not been tested *in vivo*.

In this study, we develop a method for predicting the direct competition of peptides for presentation by MHC-I. We experimentally measure the influence of varying peptide supply on the selection of two competing peptides of different off-rates, and calibrate the peptide filtering relation (Eq. 1) with these data. We also generate model predictions for how competition for surface presentation varies when the competitor peptide off-rate is varied. In doing so, we demonstrate, for the first time, that the filtering relation holds for individual peptides in direct competition with one another and show that the same level of competition can be achieved by a high concentration of a low affinity peptide or a low concentration of a high affinity peptide. We apply this model-based approach to derive a more quantitative understanding of the changes in cell surface abundance of two competing peptides as the antigen-presenting cell is exposed to interferon-γ (IFNγ), a cytokine which has a profound effect on the antigen processing and presentation pathways in infections, autoimmune diseases (33), and cancer.

## Materials and Methods

### Cells

The B6 fibroblasts cell line was produced from primary ear fibroblasts cells harvested from C57Bl/6 wild-type mice and immortalized by transfection with pSV3-neo plasmid (ATCC) encoding the SV40 T-Ag. RMA-S, TAP2-deficient mouse T cell line, and the above fibroblasts were cultured in RPMI 1640 (Lonza, Verviers, Belgium) supplemented with 10% FCS (Globepharm, Guildford, UK), 2 mM glutamine (Lonza), and 50 μM β-mercaptoethanol.

### Fab Antibodies

Plasmids expressing Fab antibodies specific for ASNENMETM-H2Db (E10) and SSLENFRAYV-H2Db (1C3) were a kind gift from J. Bennink (34). Rosetta2 (DE3) pLacI competent bacteria (Novagen) were transformed, grown to OD600 = 1–1.2 and then induced by the addition of 1 mM isopropyl b-d-thiogalactoside (IPTG) for 3 hr at 30°C. Proteins were extracted with BugBuster reagent (Novagen) and applied to a HisTrap Excel column (GE Healthcare, Uppsala, Sweden). Bound Fabs were eluted with 250 mM imidazole and then further purified by gel filtration on a 26/600 Superdex GF column (GE Healthcare). Purified peak fractions were concentrated using Amicon Ultra-15 10 kDa cut-off centrifugal concentrator (Merck/Millipore, Cork, Ireland).

### Generation of 1C3 Chimeric Monoclonal Antibody

To allow for a more efficient production of the 1C3 reagent we generated a chimeric monoclonal antibody using the Invivogen pFUSE system allowing expression in mammalian cells. VL and VH were amplified from the original plasmid (34) using primers VL_for (GTCTTGCACTTGTCACGAATTCACTTGATGTTGTGATGACTCAG) and VL_rev (GCATCTGCCCGTTTGATCTCGAGTTTGATCTCCACCTTGGTCC), and VH_for (CTTGCACTTGTCACGAATTCGGTGGAGTCTGGGGCTGAGG) and VH_rev (GGTGTCGTTTTAGCGCTGCTAGCGCTTGAGACGGTGACCAGG) respectively. VL and VH sequences were inserted respectively into pFUSE2ss-CLIg-mk and pFUSEss-CHIg-mG1 (Invivogen, Toulouse, France) using the SLIC cloning method to produce a chimeric monoclonal antibody containing a mouse IgG1 Fc fragment. Both plasmids were co-transfected into 293F cells and supernatant was harvested 1 week after transfection, clarified by centrifugation, and filtered through a 0.22 µm filter before use.

### Peptides

Peptides (listed in Table 1) (GL Biochem, Shanghai, China) were synthesized by fluorenylmethoxycarbonyl chemistry and were >95% pure by HPLC and mass spectrometry.

**Table 1:** Peptide sequences and off-rates.

Peptide	BIMAS score	NetMHC 4.0 (nM)	t_1/2_ (min)	Off-rates (s^-1^)	E10 score
ASNE**A**METM	3.4	2208.8	43	2.7 × 10^−4^	8.2
ASNENMET**A**	17	94.6	52	2.2 × 10^−4^	14.2
ASNENMET**V**	17	13.2	132	8.8 × 10^−5^	16.8
ASNENMET**L**	343	9.6	191	6.1 × 10^−5^	11.8
ASNENMET**I**	343	6.6	212	5.4 × 10^−5^	13.1
*ASNENMETM*	343	7.3	223	5.2 × 10^−5^	11.5
ASNEN**L**ETM	411	10	238	4.9 × 10^−5^	4.1
*SSLENFRAYV*	0.5	23.4	408	2.8 × 10^−5^	3.8
AS**I**ENMETM	1029	3	456	2.5 × 10^−5^	8.9
AS**I**EN**L**ETM	1235	3.6	502	2.3 × 10^−5^	1.8

*BIMAS score was determined using BIMAS (http://www-bimas.cit.nih.gov/molbio/hla_bind/) for 9mers binding to H-2Db. Equivalently, analysis was conducted using NetMHC 4.0, which returns the predicted IC50 of H-2Db binding (nM). Peptide half-lives (t_1/2_) were determined experimentally by BFA decay assays and converted into off-rates as log(2)/half-life (s). E10 score: E10 Fab binding affinity for all ASN–H2Db complexes was determined by flow cytometry surface staining of RMA-S cells pulsed with peptides (Figure S7 in Supplementary Material). The E10 score represents the MFI/1000*.

### Peptide-Expressing Plasmids

pSC11 plasmids containing Venus/mCherry-ubiquitin-peptide cassettes were obtained from J. Bennink. These plasmids were used in Ref. (35) to generate recombinant vaccinia viruses expressing the following peptides: SSLENFRAYV, PA_224–233_ and ASNENMETM, NP_366–374_. To be able to use those constructs in transient transfection assays the cassettes were recloned into pEGFP-Ub-SIINFEKL (36) (obtained from J. Neefjes). Venus/mCherry-Ub-peptide cassettes were amplified from the pSC11 plasmids by PCR using primers CGCTAGCGCTACCGGTCGCCACCATGGTGAGCAAGGGCGAG and CGCTCACAGAATTCCCAGCG containing a Nhe1 and EcoR1 restriction sites respectively (underlined). The PCR product was amplified using GoTaq Flexi DNA Polymerase (Promega) to enable ligation into the pGEM-T vector system (Promega). pGEM-T-cassette plasmid sequences were checked by sequencing using SP6 and T7 promoter primers. pGEM-T-cassette and pEGFP-Ub-SIINFEKL plasmids were then cut with Nhe1 and EcoR1 and the EGFP-Ub-SIINFEKL cassette was replaced with the Venus/mCherry-Ub-peptide cassette using the Roche Rapid Ligation Kit (Roche). Resulting plasmids were checked by sequencing using the above primers.

### Generation of ASNENMETM Variant Plasmids

The pVenus-Ub-ASNENMETM construct was used as a template to generate a series of variants by site-directed mutagenesis using the QuickChange II Site-Directed Mutagenesis Kit following the manufacturer’s protocol using the Pfu Ultra enzyme (Agilent). The list of the variants can be found in Table 1.

### Brefeldin A Decay Assay

Dissociation of pMHC complexes at the cell surface was assessed by BFA decay assay as described previously (17). RMA-S cells were incubated overnight at 26°C to maximize MHC-I surface expression. After being washed, 5 × 10^5^ cells per well of a 96-U-bottom plate were pulsed, at different time intervals, with peptides (final concentration of 20 μM) for 1 h at 26°C. After washing with medium, *de novo* transport of MHC-I to the cell surface was blocked by the addition of BFA, and peptide-loaded RMA-S cells were incubated at 37°C to allow decay of unstable molecules. Cells were washed in FACS buffer and stained with B22 primary monoclonal Abs (conformation sensitive anti-H-2Db MAb) and goat anti-mouse IgG-PE secondary Abs (Abcam, Cambridge, UK) to detect peptide-loaded MHC-I molecules. Samples were analyzed by flow cytometry on a Fortessa X20 flow cytometer (BD, Oxford, UK), and data were analyzed with the Diva software. MFI values were background deducted by subtracting the MFI value obtained in the DMSO control at the last time point. Half-lives and off-rate constants were then determined by fitting the curves using an exponential trend line in the Excel software (Microsoft, USA).

### pMHC Competition Assay

Fibroblasts were seeded at 2 × 10^5^ cells per 6 cm diameter Petri dish. When IFNγ treatment was applied, 1 µg of mouse IFNγ (Peprotech, Rocky Hill, USA) was added per Petri dish 4 h after seeding. Cells were transfected the following day with TransIT-LT1 (Mirus, Madison, USA) following the manufacturer’s recommendations using 2.5 µg of each Venus-Ub-peptide and mCherry-Ub-peptide constructs. 1 day after transfection cells were stained for 45 min on ice with primary reagents, 1C3 hybrid Mab neat supernatant, E10 purified Fab, B22, or Y3 purified Mabs to detect surface pMHC complexes. After washing, cells were incubated for 45 min with AF647-conjugated goat anti-human IgG (used after Fab primary) or goat anti-mouse (after mouse antibodies including 1C3) (Invitrogen/Molecular Probes, Eugene, USA). Flow cytometry was performed using a Fortessa X20 cytometer (BD) and the data were analyzed using FACS Diva software (BD).

### mCherry Calibration

mCherry flow cytometry calibration beads (Clontech/Takara, USA) were used, as recommended by the manufacturer, to calibrate the amount of mCherry molecules, equivalent to the number of peptide molecules expressed in the transfected cells. Beads were run through the flow cytometer using the same setup as for acquiring cells.

### Quantitation of pMHC Surface Expression by Indirect Immunofluorescence Assay Using Qifikit Calibrator Beads

The number of SSLENFRAYV-H2Db complexes presented on the cell surface was estimated using Qifikit beads (Dako, Glostrup, Denmark). Beads were treated as recommended by the manufacturer but stained with AF647-conjugated goat anti-mouse IgG (Invitrogen) at the same dilution as used to detect the 1C3 Mab on transfected cells. A calibration curve was drawn by plotting the MFI of the 5 peaks (*x*-axis) versus the lot-specific numbers of antibody molecules per bead (*y*-axis). The curve was then used to calculate the number of SSLENFRAYV-H2Db complexes presented on the cell surface expressed in antibody-binding capacity units (number of primary mouse monoclonal antibodies per cell).

### Calibrating the Peptide Filtering Model to Flow Cytometry Data

The peptide filtering model established in Ref. (32) was adapted to analyze measurements of peptide competition. The peptide filtering model is described by a system of biochemical interactions, as follows:

- Synthesis/degradation reactions :
  $$Ø\overset{g_{i}}{\underset{d_{P}}{⇌}}P_{i},Ø\overset{g_{M}+\gamma }{\underset{d_{M}}{⇌}}M,Ø\overset{10(g_{M}+\gamma )}{\underset{d_{T}}{⇌}}T$$
- Tapasin − MHC-I binding :
  $$\begin{array}{l} T+M\begin{array}{c} b_{T}\\ ⇌\\ u_{T} \end{array}TM \end{array}$$
- Peptide binding/unbinding :
  $$\begin{array}{l} M+P_{i}\begin{array}{c} b\\ ⇌\\ u_{i} \end{array}MP_{i},\;TM+P_{i}\begin{array}{c} c\\ ⇌\\ q.u_{i} \end{array}TMP_{i} \end{array}$$
- Peptide-induced tapasin dissociation :
  $$TMP_{i}\overset{\nu_{T}}{\to }T+MP_{i}$$
- Egression :
  $$MP_{i}\overset{e}{\to }MeP_{i}$$
- Peptide unbinding at the cell surface :
  $$MeP_{i}\overset{u_{i}}{\to }Me$$
- MHC-I degradation at the cell surface :
  $$Me\overset{d_{Me}}{\to }Me$$

where *i* denotes the peptide (e.g., SSLENFRAYV, ASNENMETM, or self) and Ø denotes no molecule. All parameters in the peptide filtering model were taken to be identical to those in the original publication (32), except for the on-rate for ASNENMETM, *b*_ASN_, which was allowed to differ from the nominal binding rate that was used for SSLENFRAYV. Also, a new variable γ was used to approximately quantify the increase in MHC-I and tapasin supply in IFNγ-treated cells. The peptide supply rates were defined using fluorescence measurements corresponding to intracellular peptide abundance (*F_i_*), which were multiplied by scale factors *f_i_*:

$$g_{i}=f_{i}\times F_{i}$$

To relate the output of the model to fluorescence measurements corresponding to cell surface presentation (*H_i_*), model outputs were scaled by peptide-specific scale factors *h_i_*, to give estimates

$${\hat{H}}_{i}=h_{i}\times \left[MeP_{i}\right]$$

All new parameters were inferred by fitting the simulated fluorescence measurements ${\hat{H}}_{i}$ to the experimental fluorescence measurements.

We used the Visual GEC software^1^ to perform parameter inference, which uses the domain-specific Language for Biochemical Systems (LBS) for specifying the reaction system, and an adaptive Metropolis–Hastings Markov chain Monte Carlo (MH-MCMC) algorithm from the Filzbach software.^2^ The inference of parameters in LBS models using MH-MCMC is described in the Supplementary Information of Ref. (37), but we provide a short summary here. MH-MCMC enables the calculation of the posterior distribution of the parameter values θ, given observation data *D*, and some prior belief of the parameter values. We write the posterior distribution as π (θ*|D*). The MH-MCMC algorithm uses an iterative stochastic search technique in which parameter sets are sampled in such a way that a Markov chain is formed, with the history of the chain converging to π (θ*|D*). At each iteration, the current parameter set θ is perturbed to generate a new proposed set θ*. The new parameter set is accepted or rejected based on the ratio of a likelihood function *L*(θ) evaluated at each parameter set, such that improvements are always accepted, but lower likelihoods are accepted with some probability. In this way, the chain converges toward and does random walks in regions of high probability mass, avoiding wasting time (computational effort) in regions of lower probability mass. For more details on MCMC algorithms, we recommend (38) in addition to the Filzbach software documentation.

Internally to Visual GEC, the LBS model is simulated as deterministic rate equations, and the simulation output is then related to the experimental data using the log-likelihood function

$$\log\;L\left(\theta \right)=\sum_{k=1}^{N_{d}}\log P(y_{k}|\theta ),\;y_{k}∼N\left(x_{k},\sigma^{2}\right)$$

where the *x_k_* are the model simulations, *N_d_* is the number of measurements, and σ is the SD of the measurement error, which is inferred along with the calibration parameters. As Visual GEC expects time-series measurements, the experimental data were specified at a time of 48 h, to enable the peptide filtering model to reach its equilibrium (LBS code is available from the authors upon request). During application of the MH-MCMC algorithm, the calibration parameters are varied, and there is convergence toward values that yield simulation values that are closer to the measured data, thus approximately maximizing the likelihood function.

## Results

### Surface Presentation of a Target Peptide Decreases With Increasing Amounts of Competitor

**Figure 1:**
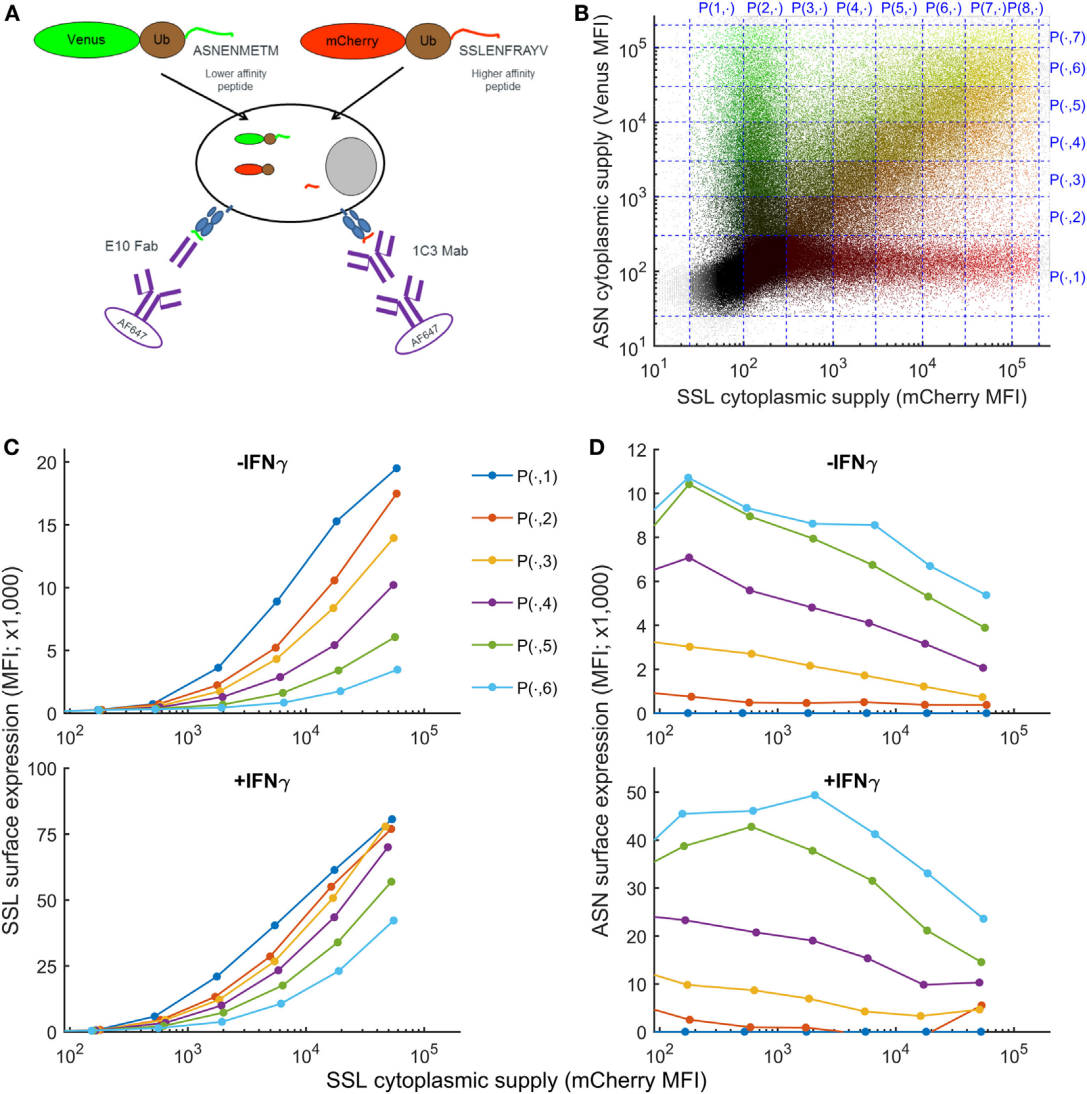
Simultaneous measurement of intracellular peptide abundance and cell surface peptide–MHC complex (pMHC). **(A)** Experimental setup. Fibroblasts were co-transfected with constructs expressing fusion proteins made of a fluorescent protein, ubiquitin, and a peptide. Cytoplasmic ubiquitin hydrolases cleave the fusion proteins, releasing an equimolar ratio of peptide and fluorescent protein. Peptides are transported to the endoplasmic reticulum where they can compete for loading onto MHC-I molecules. Then they migrate to the cell surface where ASNENMETM-H2Db complexes can be detected using E10 Fab and SSLENFRAYV-H2Db using the 1C3 chimeric Mab followed by a secondary antibody conjugated to AF647. **(B)** In a single transfection assay, cells were expressing low to high levels of both fusion proteins and were separated in different gates for the purpose of the analysis. **(C)** Level of SSLENFRAYV-H2Db surface expression in the presence of increasing amount of competitor. The dark blue curve shows the maximum surface expression as the cytoplasmic level of SSLENFRAYV peptide, represented on the *x*-axis, increases. The other curves represent the SSLENFRAYV-H2Db surface expression in the presence of different levels of ASNENMETM competitor [top dark blue curve corresponds to gates P(1, 1) to P(8, 1) with no competitor, down to the light blue bottom curve corresponding to gates P(1, 8) to P(8, 8) with the maximum level of competitor] in untreated wild-type cells (top panel) or in IFNγ-treated cells (bottom panel). **(D)** Corresponding ASNENMETM-H2Db surface expression.

To establish how variation in the intracellular abundance of competing peptides influences cell surface presentation, we developed an assay in which intracellular peptide supply and pMHC cell surface abundance could be measured simultaneously (Figure 1A), assay adapted from Ref. (35). Furthermore, this method measures the abundance of peptides actually presented at the surface of living cells and not, as for quantitative high-throughput methods, the abundance of peptides remaining bound to MHC after biochemical purification of pMHC I complexes from cell lysates. Two fluorescent fusion proteins, Venus-ubiquitin-ASNENMETM (faster off-rate, see Table 1) and mCherry-ubiquitin-SSLENFRAYV (slower off-rate) are co-expressed in fibroblasts. Once expressed in the cytoplasm, the fusion proteins are cleaved by endogenous cytoplasmic ubiquitin hydrolases, releasing the peptides at an equimolecular ratio to their respective fluorescent reporter protein (Figure 1A) that can be quantified by flow cytometry (36) (Figure S1 in Supplementary Material). Using this system, the generation of peptides bypasses the proteasome. After translocation into the ER, peptides compete for loading onto MHC-I molecules and are transported to the cell surface where they can be quantified by flow cytometry (Figure 1A). Both fusion proteins were naturally expressed at a broad range of concentrations after transient transfection, allowing in a single experiment to compare peptide surface presentation in cells expressing low to high levels of both fusion proteins (Figure 1B). To analyze competition between both peptides, cells were partitioned into different gates according to their expression level of mCherry, reporter for the level of expression of SSLENFRAYV, and Venus, reporter for the level of ASNENMETM (Figure 1B). Competition was then assessed by plotting surface expression of SSLENFRAYV-H2Db as a function of increasing cytoplasmic expression of the SSLENFRAYV target peptide for increasing levels of competing peptide (Venus levels 1–7 in Figure 1B). Figure 1C (top panel) shows that surface expression of the slower off-rate SSLENFRAYV (2.8 × 10^−5^ s^−1^) decreased as expression of the faster off-rate ASNENMETM (5.2 × 10^−-5^ s^−1^) increased from P(.,1) to P(.,6). Simultaneously, ASNENMETM (Figure 1D, top panel) became more abundant on the cell surface.

To determine the order of magnitude of the inferred parameters for our previously published model (32) we quantified the supply of the target peptide (*g*_SSL_). We considered that the number of SSLENFRAYV target peptides expressed in the cytoplasm was proportional to the number of mCherry molecules, using the simplifying assumptions that all of the fusion proteins are cleaved and that the degradation rate of the fluorescent protein equals the degradation rate of the peptide. This number was determined using mCherry calibration beads (Figure S1 in Supplementary Material) and indicated that transfected cells expressed up to 10^8^ copies of SSLENFRAYV per cell and that surface expression could be detected when cytoplasmic expression approached 10^6^ copies per cell (Figure S1E in Supplementary Material). Venus expression could not be calibrated in the same way due to the lack of available reagents.

### Quantification of the Number of Target pMHC Complexes on the Cell Surface

The number of SSLENFRAYV pMHC presented on the cell surface (*MeP*_SSL_) was then quantified using Qifikit calibration beads, coated with well-defined quantities of monoclonal antibodies mimicking cells with different antigen densities bound to a primary antibody (Figure S1 in Supplementary Material). In untreated cells, up to 80,000 pMHC complexes were presented on the cell surface in the absence of ASNENMETM competition, and that number was reduced to 10,000 or less in the presence of the highest level of competitor (Figure S1E in Supplementary Material). These values are consistent with the level of expression of abundant peptides measured previously. For example, between 70,000 and 80,000 SIINFEKL–H2Kb complexes were observed on L-Kb cells infected with VV-SIINFEKL recombinants using the same Qifikit assay (39); around 45,000 copies of a human peptide (AETPDIKLF) derived from the RS5 protein were eluted from B44:02 complexes on B lymphoblastoid cell lines (25); some 32,000 copies of the A47_138–146_ vaccinia peptide were eluted from DC2.4 cells infected with vaccinia WR (4) and *ca* 24,000 copies of the most abundant peptide were eluted from B-LCL-JY pp65 cells (40). Thus our assay and model are consistent with physiological values.

### Surface Presentation of Two Competing Peptides Is Enhanced in the Presence of IFNγ

IFNγ is known to increase expression of MHC-I, together with chaperones involved in antigen processing and presentation, and plays an important role in inflammatory immune responses to viruses and cancer. However, it is not known whether IFNγ enhances presentation of all peptides or focuses the immune response on a selected few. To model the effect of IFNγ on peptide presentation, we generated an equivalent dataset for the simultaneous presentation of SSLENFRAYV and ASNENMETM on cells treated with IFNγ for 48 h. Surface expression of total H-2Db and H-2Kb (Figure S2 in Supplementary Material) as well as both SSLENFRAYV and ASNENMETM complexes increased around fourfold (Figures 1C,D), in agreement with the increase in the number of MHC-I molecules available for binding a peptide. The presentation of SSLENFRAYV was less inhibited by ASNENMETM even at high expression levels (Figure 1C bottom panel), such that less competition was observed. For example, when mCherry-SSLENFRAYV was expressed at around 10^4^ MFI units, yielding a cell surface abundance of around 4 × 10^4^ complexes in the absence of competitor, around ten times more ASNENMETM expression was required to inhibit its presentation by 50% in the presence of IFNγ compared to non-IFNγ-treated cells (where 25 × 10^4^ copies were expressed at the cell surface in the absence of competition) (Figures S1E,F in Supplementary Material).

### A Calibrated Mechanistic Model Explains Experimental Observations of Peptide Competition

Our previously published model (32) describes the endogenous antigen presentation pathway from the point where peptides are supplied to the ER, through presentation at the cell surface. The model also explicitly describes the interaction between MHC and tapasin, incorporating the binding of peptides to MHC–tapasin complexes, that influences peptide loading.

**Figure 2:**
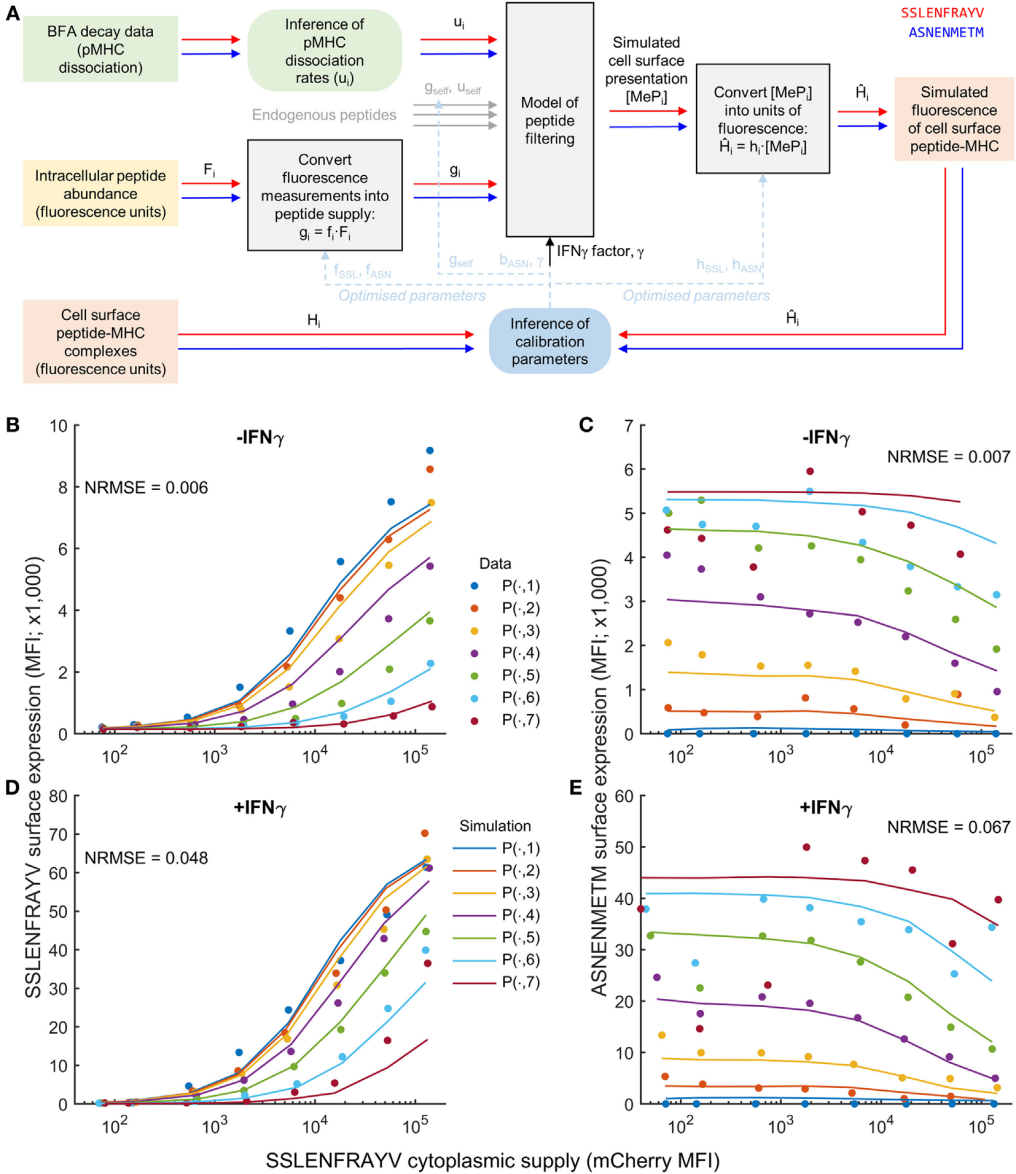
Calibration of a mechanistic model of peptide competition. **(A)** Diagrammatic representation of the process of model calibration. The cytoplasmic concentration of each peptide and their MHC unbinding rate were used as inputs to the model, which was then fit to fluorescence data of the pMHC complex surface abundance. Square colored boxes indicate measured or simulation data, gray boxes indicate models, and rounded boxes represent inference algorithms. The red and blue line connectors represent peptide-specific information, and the dashed blue lines indicate that inferred parameters are eventually substituted back into the models for simulation/prediction. **(B–E)** Comparison of the model (solid lines) evaluated at the maximum likelihood parameter values against experimental measurements (circles) for a single experiment measuring SSLENFRAYV/ASNENMETM competition. The different colors represent different cytoplasmic levels of ASNENMETM, as shown in Figure 1. The normalized root mean square error (NRMSE) between the data and the simulation is also indicated for each comparison. An equivalent comparison for a second experiment that was also included in the parameter calibration is shown in Figure S6 in Supplementary Material.

To test the extent to which peptide competition could be predicted based on both peptide affinity and intracellular abundance, we adapted the peptide filtering model of Ref. (32). To relate the model directly to the fluorescence measurements corresponding to intracellular peptide abundance and cell surface abundance in Figure 1, we transformed the experimental data from units of fluorescence into units of molecule numbers using calibration parameters (Figure 2A). For peptide supply, we specified the parameters of the model to be proportional to the intracellular abundance measurements, with proportionality factors *f*_SSL_ and *f*_ASN_ (see Materials and Methods). These factors incorporate the conversion from fluorescence units into numbers of peptides, but also implicitly account for any differences in TAP translocation. To compare the model output with the cell surface fluorescence measurements, a similar strategy was used, whereby two scale factors converted from numbers of cell surface pMHC into equivalent measured fluorescence (*h*_SSL_ and *h*_ASN_; see Materials and Methods). In addition to the target and competitor peptides, we also included a third peptide in the model to represent the presence of self-peptides in the system (Figure 2A), with a pMHC unbinding rate *u*_self_, and ER supply rate *g*_self_.

We experimentally measured the stability of pMHC complexes by following their decay from the cell surface over time (“*BFA decay assay*” in Figure 2A), and used these measurements to calculate the corresponding unbinding rates of the peptides from MHC (Table 1; Figure S3 in Supplementary Material). Finally, to incorporate the effect of IFNγ into the model, we specified a new parameter (γ) that quantifies the increased supply of MHC and tapasin following IFNγ treatment.

To establish estimates for the parameters, we used MCMC parameter inference (see Materials and Methods for further details) applied to two experimental datasets measuring intracellular and cell surface abundance of a target peptide SSLENFRAYV and competitor peptide ASNENMETM. Two repeated peptide competition experiments between SSLENFRAYV and ASNENMETM were used to infer the model calibration parameters. This enabled us to account for inter-experiment variations in the measurement of SSLENFRAYV-H2Db at the cell surface using the 1C3 monoclonal antibody. Accordingly, *h*_SSL_ and γ were allowed to take on different values between the two experiments, whereas the other parameters were assumed to be invariant. To obtain a good fit to the data, and establish robust estimates of all parameters, we made two additional changes to the setup described thus far: (i) a better fit to the data could be obtained when allowing the peptide on-rate for ASNENMETM to take on a different value from SSLENFRAYV and (ii) we found that both *u*_self_ and *g*_self_ could not be identified uniquely, so we assumed a value of *u*_self_ = 10^−4^ s^−1^, representing a peptide of average affinity for MHC-I, and inferred *g*_self_.

The results of the MCMC procedure illustrate only moderate uncertainty in the parameter estimates (Figure S4 in Supplementary Material), and also low pairwise correlation (Figure S5 in Supplementary Material). This suggests that the parameters are well constrained by the data. When parameters are not well constrained by data, there can be flexibility in assigning parameter values, for instance changes in one parameter can be compensated for by changes in another parameter. When this happens, the likelihood function (which quantifies the goodness of fit to the data) will be equally high over a structured region in the parameter space, leading to strong correlation and broad marginal posterior distributions. As such, plots of the pairwise correlations and marginal posterior distributions are commonly used as a diagnostic in Bayesian parameter fitting.

Simulation of the resulting maximum likelihood parameter set displayed a reduced presentation of each peptide in response to increasing abundance of the other (Figures 2B,C; Figure S6 in Supplementary Material), as observed experimentally. Furthermore, our hypothesized increase in MHC-I and tapasin supply in IFNγ-treated cells led to increased simulated presentation of both peptides, and a reduction in the effect of competition (Figures 2D,E), as observed experimentally.

### The Model Predicts the Effects of Different Competitors on the Surface Presentation of a Target Peptide

**Figure 3:**
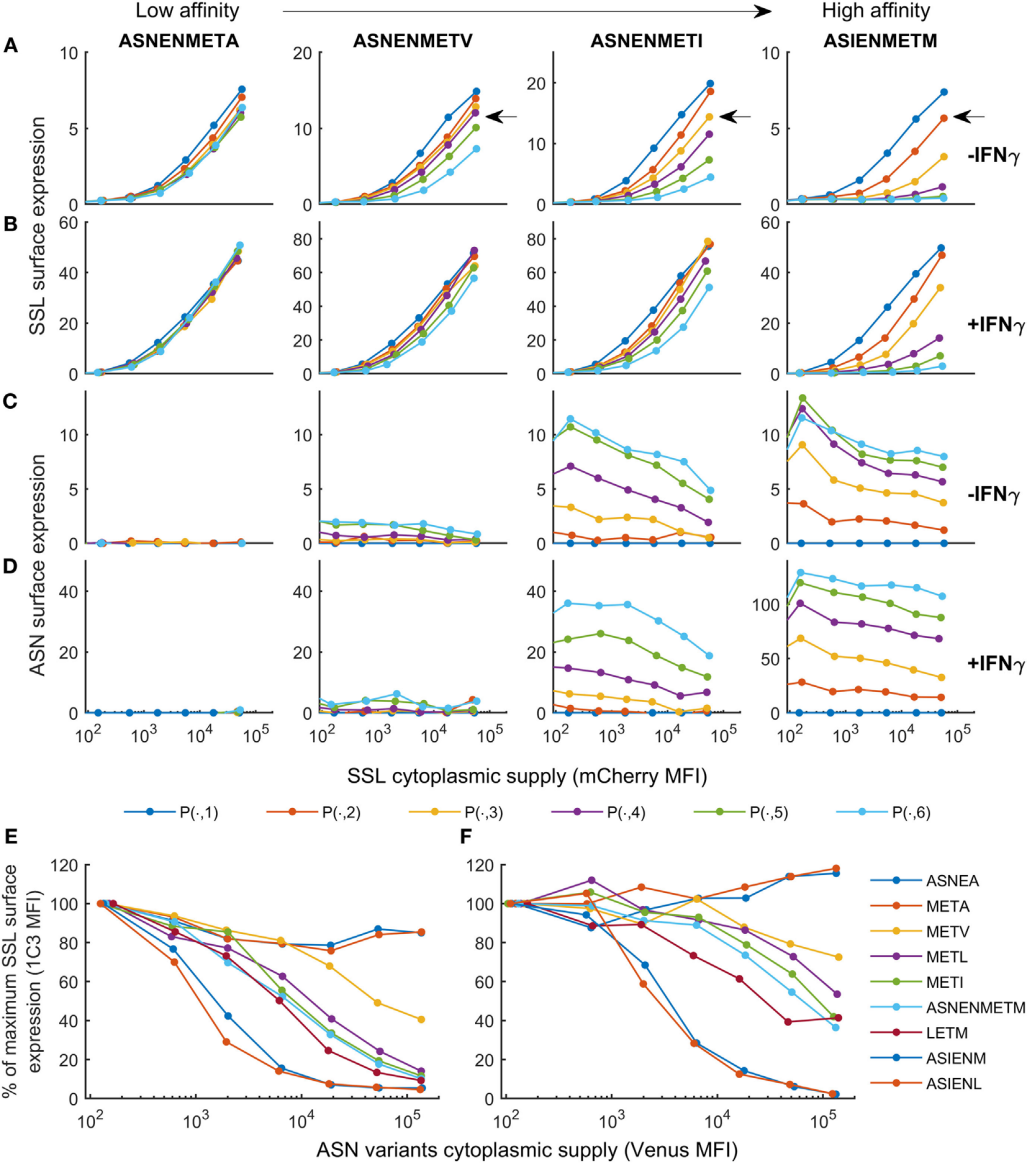
Cell surface abundance is differentially affected by competitor peptides with varying major histocompatibility complex stability. SSLENFRAYV-H2Db surface expression in the presence of ASN-variant peptides of different off-rates in untreated cells **(A)** and in cells treated with IFNγ **(B)** and corresponding ASN variants-H2Db surface expression in untreated cells **(C)** or in cells treated with IFNγ **(D)**. pMHC surface expression is plotted over the level of SSLENFRAYV peptide cytoplasmic supply, determined by the mCherry MFI, in the presence of increasing concentrations of the ASN variant peptides: the dark blue curve is obtained with no ASN variant expression [gates *P*(*n*, 1)], the orange curve corresponds to gates *P*(*n*, 2), etc. The arrows on the top graphs show a similar competition level achieved with a high concentration of a lower affinity peptide (ASNENMET**V**) or lower concentrations of higher affinity peptides (ASNENMET**I** and AS**I**ENMETM). **(E,F)** Comparison of the level of competition in the presence of an increasing concentration of each ASN-variant peptide at constant SSL supply [gates *P*(7, )]. In order to normalize MFI between experiments the *y*-axis represents the percentage of maximum SSLENFRAYV-H2Db surface expression in *P*(7, 1). The *x*-axis represents the cytoplasmic expression levels of each ASN variant expressed as the Venus MFI. Untreated cells **(E)** and IFNγ-treated cells **(F)** were compared.

The purpose of the calibrated model is to predict surface presentation of a target peptide in the presence of competitors of different off-rates. In order to test predictions made by the model, surface expression of SSLENFRAYV was measured experimentally in the presence of competitor peptides of different off-rates. Using peptide binding prediction tools (BIMAS and NetMHC 4.0), we selected ASNENMETM variant peptides which should have a range of affinities either lower or higher than the original peptide (Table 1). Off-rates were then determined experimentally in brefeldin A decay assays and comparison to the predicted values showed that in this case BIMAS performed better than NetMHC (Table 1). The corresponding plasmids encoding each Venus-Ub-variant were generated and used in competition assays. Surface expression of ASNENMETM and its variants was determined in parallel by staining with E10 Fab (Figures 3C,D). This was only possible for variants of ASNENMETM that were recognized well by E10 (Figure S7 in Supplementary Material; Table 1) and precluded variants with leucine at position 6.

Figure 3 shows the experimentally determined surface presentation of SSLENFRAYV and four measurable ASNENMETM variants in the presence (Figures 3B,D) and absence (Figures 3A,C) of IFNγ; as well as the presentation of SSLENFRAYV in the presence of increasing intracellular expression of all nine Venus-Ub-ASN(variant) constructs in the presence (Figure 3F) and absence (Figure 3E) of IFNγ. It shows a trend of increased competition with SSLENFRAYV for H-2Db binding and presentation as the stability of the variants increased, with maximum competition observed in the presence of the slowest off-rate peptides AS**I**ENMETM and AS**I**EN**L**ETM (off-rates of 2.5 × 10^−5^ s^−1^ and 2.3 × 10^−5^ s^−1^ respectively; Table 1; Figures 3A,E). However, the same level of competition could be achieved by a low concentration of a high affinity competitor or by a higher concentration of a low affinity competitor, emphasizing the importance of both peptide stability and intracellular abundance in determining cell surface presentation (Figure 3A, black arrow).

In IFNγ-treated cells, in all cases, surface expression of SSLENFRAYV was less affected by the competitors than without IFNγ (Figures 3B,F) and a higher surface expression of the competitors was also observed for the slower off-rate peptides (Figure 3D). IFNγ did not, however, enhance surface expression of the fast off-rate variants (ASNE**A**METM, ASNENMET**A**, and ASNENMET**V**) which are still unable to reach or remain at the cell surface at detectable levels.

**Figure 4:**
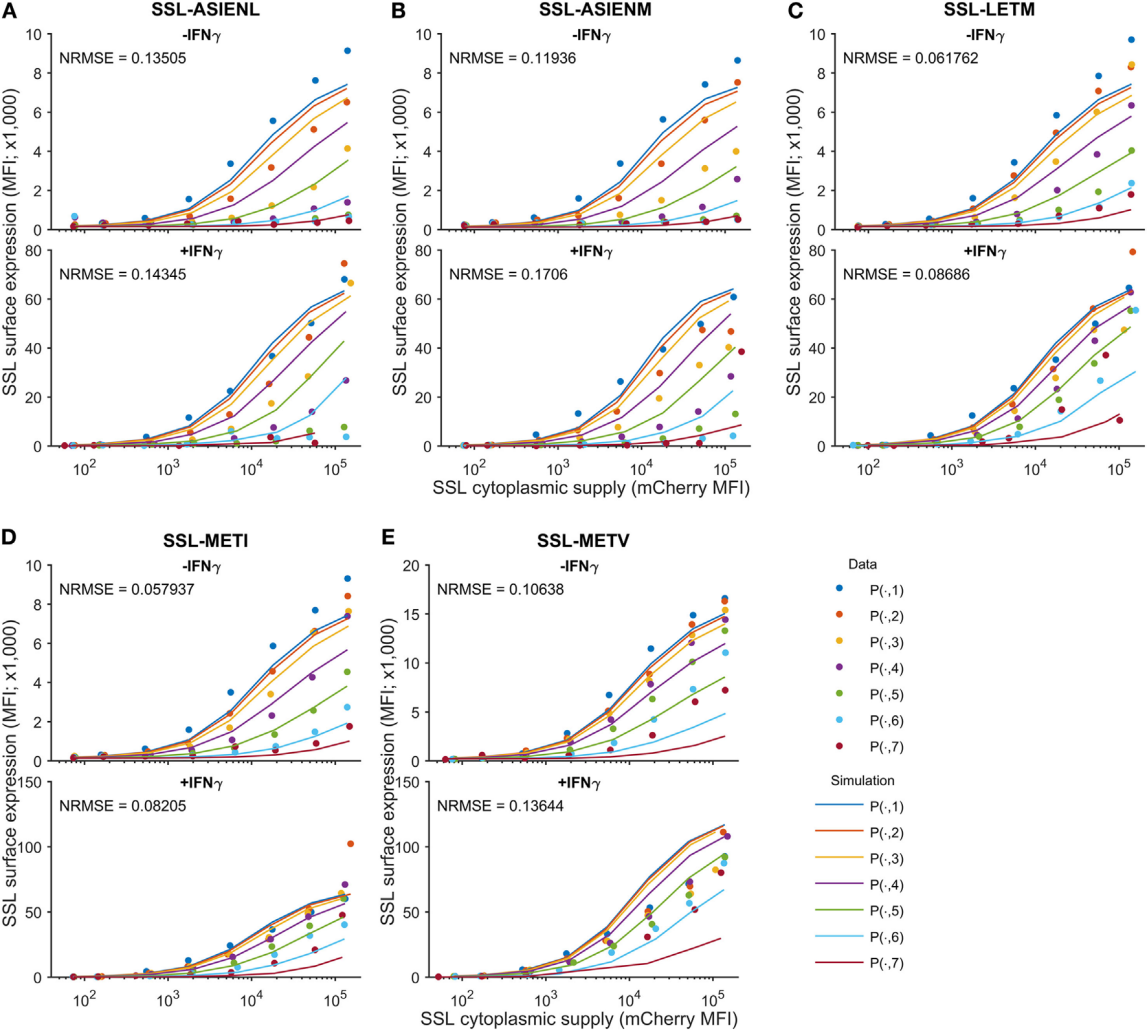
Predicting cell surface presentation between peptide competitors. Using the calibrated model, cell surface presentation was predicted for SSLENFRAVY competing against variants of the ASNENMETM peptide, by changing the peptide off-rates to the measured values in Table 1. Shown are predictions and measurements for cell surface presentation of SSLENFRAYV, both in untreated (top panels) and IFNγ-treated (bottom panels) cells, when competing against ASN variant peptides **(A)** ASIENLETM, **(B)** ASIENMETM, **(C)** ASNENLETM, **(D)** ASNENMETI and **(E)** ASNENMETV. Predictions of the cell surface presentation of ASNENMETM variants are shown in Figure S8 in Supplementary Material. Different colored traces represent different cytoplasmic levels of the competing ASNENMETM variant, as indicated in Figure 1B. The normalized root mean-squared error (NRMSE) between the data and the simulation is also shown for each panel.

We next sought to determine the predictability of peptide competition, based on a quantitative knowledge of cytoplasmic abundance and off-rate from MHC-I. Using the calibrated peptide filtering model, we predicted the cell surface presentation of SSLENFRAYV pMHC when competing against variants of the ASNENMETM peptide, by simulating with their measured off-rates (Table 1). To control inter-experiment variability, the competition experiments were carried out at the same time as a SSLENFRAYV-ASNENMETM competition experiment. We therefore used the corresponding MHC-I and tapasin supply rates inferred for these experiments during model calibration. The Venus fluorescence intensity can be used to compare corresponding levels of expression of the different ASNENMETM variants as they are all expressed in the cytoplasm at the same ratio of 1 peptide per molecule of Venus. We assumed that ASNENMETM variants bind to TAP with similar affinities and, therefore, that their rate of ER entry is similar. As the hierarchy of competition follows the hierarchy of peptide stability, this assumption is reasonable. The resulting predictions are superimposed on the corresponding experimental measurements in Figure 4. The model predictions display the same characteristics as the data: increasing abundance of the competing variant of ASNENMETM leads to an increase in its own cell surface presentation (Figure S8 in Supplementary Material) but a drop in SSLENFRAYV presentation (Figure 4), and IFNγ treatment increases presentation of both peptides. However, similar to the model output for SSLENFRAYV presentation when competing with ASNENMETM in IFNγ-treated cells (Figure 2D), only a modest reduction in competition could be seen in predictions of competition against ASNENMETM variants (Figure 4).

### Cell Surface Abundance Is Accurately Predicted by a Peptide Competition Metric

While the full peptide filtering model is demonstrably capable of reproducing and predicting measurements of peptide competition across a range of peptides, the complexity of the model does not offer a simple quantitative explanation. Therefore, we considered whether competition could be explained by the filter relation [(32); see above], which approximates equilibrium cell surface presentation of a peptide in terms of its supply and off-rate from MHC-I. To incorporate a contribution from the competitor peptide, we investigated a normalization of the filter relation (Eq. 1) as

(2) $$MeP_{\text{ratio}}\approx \frac{{\left[P_{\text{SSL}}\right]}_{\text{cyt}}/u_{\text{SSL}}{\;}^{2}}{{\left[P_{\text{ASN}}\right]}_{\text{cyt}}/u_{\text{ASN}}{\;}^{2}+g_{\text{self}}\text{/}u_{\text{self}}{\;}^{2}}$$

where SSL denotes the SSLENFRAYV peptide, ASN denotes an ASNENMETM variant, and *MeP*_ratio_ represents the ratio of egressed SSL complexes to other complexes (ASN and self). ${\left[P_{k}\right]}_{\text{cyt}}=f_{k}.F_{k}$ denotes the calibrated cytoplasmic abundance of peptide *k* [i.e., *k* is either SSL or ASN; the scaling factor *f_k_* converts units of fluorescence *F_k_* into number of molecules (*P_k_*)_cyt_]. In this definition (Eq. 2), the absence of competitor peptides leads to surface presentation of SSLENFRAYV being given purely in terms of the background self-peptides.

We calculated the peptide competition metric (Eq. 2) for each peptide competition experiment and compared the output against the corresponding simulations of the peptide filtering model. This established the competition metric to be a good approximation of the equilibrium behavior of the full model, both in the untreated (Figure S9 in Supplementary Material) and IFNγ-treated regimes (Figure S10 in Supplementary Material). Variations in the abundance of the competitor peptides could be almost entirely accounted for by the metric, which can be seen in Figures S9 and S10 in Supplementary Material as the different colored traces (corresponding to different levels of competitor abundance) collapsed onto a consistent relationship between the metric and model-predicted cell surface abundance of SSLENFRAYV-H2Db.

**Figure 5:**
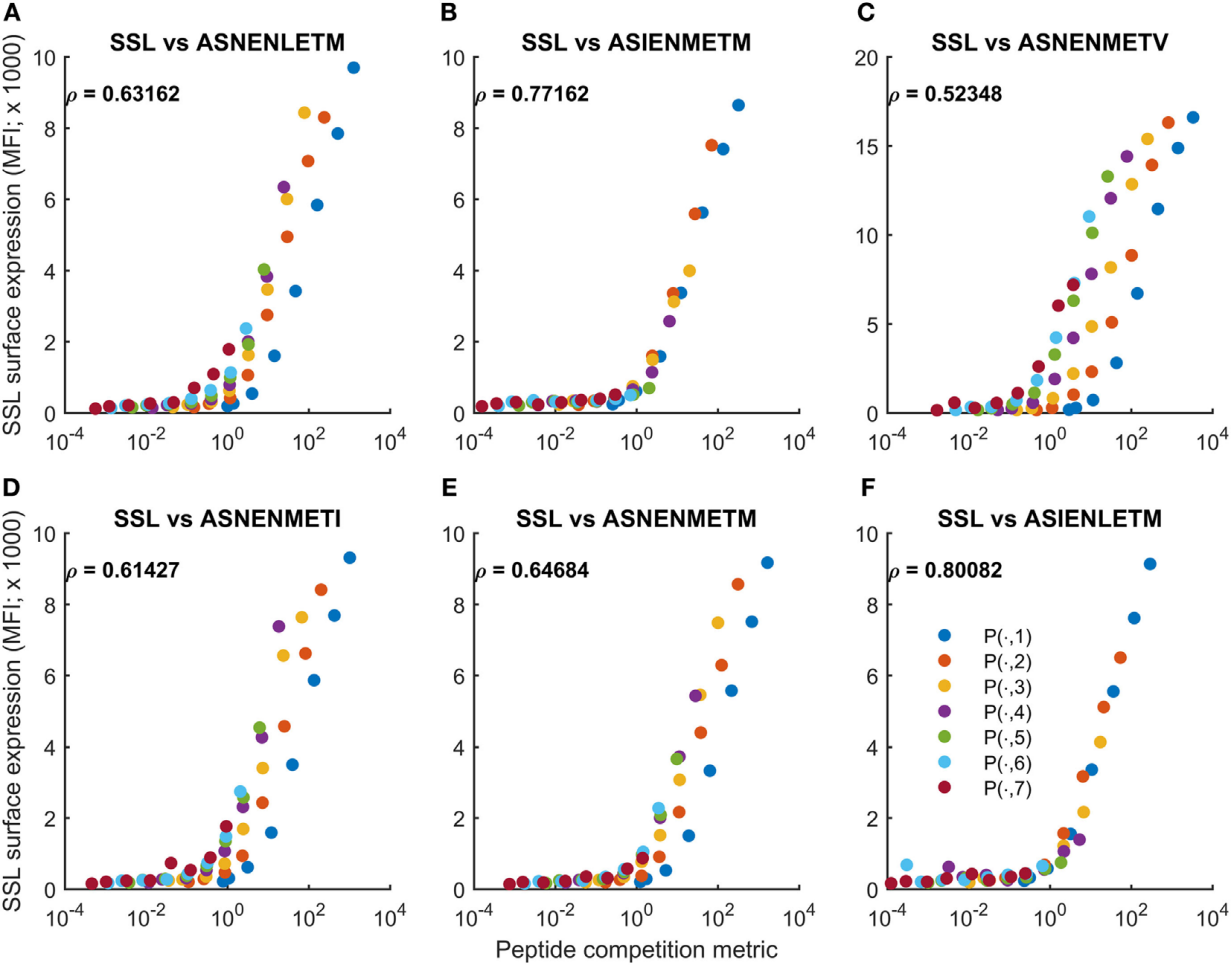
Cell surface abundance can be predicted by a peptide competition metric. The peptide competition metric *F*_ratio_ (Eq. 3) was calculated using measurements of cytoplasmic peptide abundance for SSLENFRAYV and variants of ASNENMETM (fluorescence units in Figures 1 and 3). *F*_ratio_ (horizontal axis) was then compared with experimental measurements of cell surface abundance of SSLENFRAYV (vertical axis). Different colored traces represent different cytoplasmic levels of the competing ASNENMETM variant, as indicated in Figure 1B. A to F represent competitions between SSLENFRAYV and ASNENLETM **(A)**, ASIENMETM **(B)**, ASNENMETV **(C)**, ASNENMETI **(D)**, ASNENMETM **(E)** and ASIENLETM **(F)**.

We then applied the peptide competition metric directly to the experimental observations, without using the model. Accordingly, we calculated

(3) $$F_{\text{ratio}}\approx \frac{F_{\text{SSL}}\text{/}u_{\text{SSL}}{\;}^{2}}{F_{\text{ASN}}\text{/}u_{\text{ASN}}{\;}^{2}}-$$

where *F*_SSL_ and *F*_ASN_ represent measurements of intracellular peptide abundance (fluorescence intensity units). Unlike for *MeP*_ratio_, the metric does not include a contribution from self-peptides, as in general this quantity would not be available. We compared *F*_ratio_ with measurements of cell surface abundance of SSLENFRAYV (Figure 5). We found that variations in peptide abundance could be predicted with high accuracy for untreated cells (−IFNγ; Figure 5), though the variations in the abundance of ASNENMET**V** peptide were only partially accounted for (Figure 5C). By contrast, the peptide competition metric was less accurate for IFNγ-treated cells (Figure S11 in Supplementary Material). This is reflected in the Pearson correlation scores (compare Figure 5 with Figure S11 in Supplementary Material). Overall, we found that differences in competitor abundance are not as well captured by *F*_ratio_ as they are by *MeP*_ratio_ (Figure S10 in Supplementary Material). In particular, *F*_ratio_ over-approximates surface presentation of SSLENFRAYV when competitor peptide abundance is low. As the same over-approximation was not seen in the comparison of *MeP*_ratio_ and simulated surface abundance of SSLENFRAYV (+IFNγ; Figure S10 in Supplementary Material), our interpretation is that this is due to *F_ratio_* not incorporating the potential impact of self-peptides. At low competitor abundance, self-peptide availability will be more important, leading to a loss of accuracy of *F*_ratio_. Nevertheless, we have found that very simple formulae can largely predict semi-quantitatively how the presentation of a given peptide will be reduced by the increased abundance of a competitor peptide.

## Discussion

We have developed a mathematical model based on known cellular mechanisms which, despite including only three components (MHC, peptide, and tapasin), can predict pMHC surface expression under physiological conditions, given some knowledge of the intracellular abundance of peptides. The abundance of specific pMHC on antigen-presenting cells can determine both the intensity of the primary CTL (cytotoxic T lymphocyte) response to that pMHC and also the susceptibility of target cells bearing the pMHC to killing by those CTL. At present there is no predictive model for estimating the rate of CTL killing as a function of pMHC abundance. Current high-throughput methods for detecting MHC-I bound peptides have advanced significantly over the past decade and have given rise to better algorithms for estimating whether a particular peptide is likely to be presented as a pMHC. Nevertheless, the estimates are not quantitative and depend on the ability to detect specific peptides following stringent purification of pMHC prior to peptide extraction during which time peptides are progressively lost according to their individual dissociation rate constants. Our model relates intracellular peptide abundance to cell surface abundance *via* the intracellular process of chaperone assisted peptide editing of MHC-I, which occurs in the face of competition between millions of peptides for binding to the same MHC-I. Such conditions are likely to be especially important, for example, during viral infection where viral epitopes need to compete with a vast number of self-peptides, or when trying to generate an immune response against a polytope vaccine (multiple epitopes artificially joined into a single polypeptide, possibly being expressed from large virus vectors generating themselves many other viral epitopes), or against cancer neo-epitopes competing against a multitude of more abundant or higher affinity self-peptides.

Thus, we developed a competition assay that allowed us to measure surface abundance of a target peptide in the presence of increasing amount of competitor peptides of different off-rates. Our results highlighted the importance of the abundance of competing peptides, as indeed the same level of competition could be achieved by a low abundance of a high affinity competitor or a high abundance of a low affinity peptide. Based on these experimental data, we calibrated our model of MHC-I presentation, which was originally developed to explain how peptide optimization differs between MHC-I alleles (32). Here, we found that this same model was able to predict surface expression of a target peptide in the presence of competitors of different off-rates (Figure 4). Furthermore, we found that simple peptide competition metrics (Eqs 2 and 3), based on the filter relation (Eq. 1), could quantify the impact of increasing intracellular competitor abundance on target peptide surface presentation (Figure 5). This is the first time that the filter relation has been experimentally tested on competing peptides. The present study provides justification for its use in vaccine design, for instance, to assess the relative merits of increasing peptide abundance or improving peptide stability to achieve a desired level of cell surface presentation, using a simple calculation.

We were also able to predict pMHC surface expression when MHC-I and tapasin supply increased following IFNγ treatment, by extending the basic model to incorporate a factor quantifying the extent of IFNγ upregulation of MHC-I and tapasin. IFNγ is well known to stimulate the immune system, increasing expression of MHC-I heavy chain, β2-microglobulin, subunits of the immunoproteasome (MECL1, LMP2, and LMP7), TAP, tapasin, the ER aminopeptidase associated with antigen processing (ERAAP) (41) and the tapasin-related protein (TAPBPR); it is produced as part of the immune response against viruses. IFNγ also plays a critical function in cancer immunosurveillance (42) as it can be secreted in the tumor micro-environment, determining the inflammatory status of the tumor (43) and influencing tumor prognosis. For many years, IFNγ has been used in the clinic as an immunostimulant within immunotherapy regimes although the details of how it works were poorly understood. Those studies resulted in variable outcomes, sometimes detrimental to the patients (44). In the current study, as both the target and competitor peptides were generated from cleavage by cytoplasmic Ub-hydrolases, we were able to bypass the effect of IFNγ on the proteasome [IFNγ induces a switch from constitutive to immunoproteasome with different cleavage specificity that can be observed at the level of the immunopeptidome (45)] and only consider the effect on the antigen presentation machinery. We have confirmed in our system an increase of MHC-I and tapasin expression after treatment with IFNγ and observed an increase in surface expression of both competing peptides. The level of competition of the SSLENFRAYV peptide was reduced in the presence of IFNγ, presumably due to the increased level of available MHC-I molecules. This is in line with observations made by others, for example, (33) showed that after treating NIT-1 insulinoma cells with IFNγ, presentation of the high affinity JAK-1_355–363_ (SYFPEITHI) peptide by H-2Kb was increased from ~2,000 to ~15,000 copies per cell; whereas, the lower affinity IGRP_206–214_ peptide (VYLKTNVFK), barely detectable at 1 copy per cell in untreated cells, reached 25 copies per cell after IFNγ treatment. The differential enhancement of presentation by IFNγ (7.5- or 25-fold increase respectively) did not merely follow the fivefold increase in overall H-2Kd surface expression. This means that the peptide repertoire presented by β-cells shows subtle differences under inflammatory conditions even though the length or binding affinity of peptides presented by either H-2Kd or H-2Db did not change. In other words, cytokine treatment did not bias toward high-affinity ligands. In this instance such a subtle change in the peptide repertoire might be related to the transition from benign to destructive insulitis.

Likewise, IFNγ can also have a dramatic effect in cancer immunology. The tumor environment is likely to progress from an inflammatory surrounding with active T cells producing IFNγ, to a non-inflammatory environment populated by regulatory T cells and exhausted T cells that have stopped producing the cytokine. Tumor cells can also evolve immune escape mechanisms blocking the IFNγ pathway. It would be, therefore, beneficial to be able to predict CD8+ T cell targets that are likely to be presented on tumor cells in both presence and absence of IFNγ.

We showed in our study that the surface expression of the lower affinity peptide was enhanced in the presence of IFNγ. This could result in a CD8+ T cell response to develop against a broader range of peptides as priming, activation of a T cell at first encounter with its target pMHC, only occurs above a threshold antigen dose (1). As a result, competition between different specificity CD8+ T cells resulting in immunodomination, occurring very early on during the immune response (46), might be altered in the presence of IFNγ.

Our observations of increased intracellular abundance enhancing cell surface presentation of low affinity peptides might explain why in other systems some low affinity peptides (that lie outside the 500 nM cut-off often used to define MHC-I-binding) can be presented efficiently and are able to induce strong CD8+ T cell responses. For example, the two immunodominant epitopes from the transplantable murine tumor CT26 have half-lives of 60 and 20 min [H-2Ld binding SPSYVYHQF and H-2Dd binding GGPESFYCASW respectively (47)], despite there being almost 500 neo-epitopes generated from point mutations that are considered likely to be immunogenic as they have an IEDB percentile rank less than or equal to 1% [(48); Table S3 in Supplementary Material]. Both of these peptides originate from the highly abundant gp70 retrovirus envelope protein encoded by a gene located in a CT26 tetraploid region and transcribed at high copy number (48). It seems, therefore, that lower affinity peptides with moderate half-lives can still be presented in sufficient abundance at the cell surface to induce immunodominance. Interestingly, strong CTL responses to the GGPESFYCASW-H2Dd complex correlate with anti-tumor efficacy in immunotherapeutic settings such as Tregs depletion (47) and anti-PD-1 checkpoint blockade (G. Sugyarto, personal communication).

Mechanistic modeling has the advantage of incorporating knowledge of the antigen processing pathway and of being modifiable as that understanding grows. For example, although the current model incorporates the functions only of MHC-I and tapasin, extensions of our model could incorporate the function of ERAAP antigen processing, calreticulin (which recycles empty MHC-I from ERGIC to ER), and the emerging function of TAPBPR [which also functions as a peptide editor in connection with the UDP-glucose:glycoprotein glucosyltransferase (49)]. Our model could also be used to simulate peptide presentation in tumor escape mutants with altered expression of: (i) some of the immunoproteasome sub-units (LMP2 and LMP7) that would maintain a “non-inflammatory” peptidome even in the presence of IFNγ; (ii) proteins involved in the IFNγ signaling pathway (50) also resulting in the presentation of a “non-inflammatory” immunopeptidome within a pro-inflammatory tumor microenvironment; (iii) mutations affecting the expression of antigen processing and presentation molecules, including TAP, tapasin, and ERAAP. These mutations would result in a modification of the peptide repertoire presented by MHC-I molecules and also in a drastic reduction of the pMHC surface expression level. Low tapasin expression has been shown to correlate with low T cell infiltration and poor prognosis in colorectal cancer (51) together with the loss of presentation of some, normally, immunodominant CTL epitopes (17, 52, 53). However, tapasin expression can also have a negative impact on the presentation of other immunodominant epitopes such as MUC1 which is revealed when tapasin is downregulated (54). Unfortunately, the low level of pMHC expression in tapasin deleted cells precludes the experimental measurement of cell surface peptide abundance in these conditions. Modeling, however, could be used to predict shifts in the immunopeptidome resulting from the selective downregulation or loss of tapasin from cancer cells and may, therefore, help to guide the selection of anti-cancer vaccines or other therapies.

While we have observed how intracellular peptide abundance can influence cell surface presentation in a direct assay, the challenge will be to test our approach at the whole immunopeptidome level. However, the validation of such a model is restricted by the experimental methods available today: in particular, limitations on the biochemical isolation of peptides recovered from MHC-I mean that around 90% of the immunopeptidome is lost prior to analysis (27). Also, only a quantitative immunopeptidome generated from elution of surface pMHC would allow to demonstrate the benefit of our model including tapasin filtering versus the use of algorithms based purely on the amino acid sequence of the peptide. Another limitation is that our model requires prior quantification of intracellular peptide abundance for each peptide to produce a prediction of the cell surface presentation profile. Prediction of an entire cell surface peptide repertoire would, therefore, require high-throughput measurements of protein expression and turnover by quantitative proteomics (SILAC) (55), or measurements of transcription levels or protein translation rates, combined with proteasomal cleavage (56) and TAP binding (57) predictions. The dynamical modeling approach that we advocate (26, 58) has the advantage of encoding mechanistic hypotheses, which should enable us to also predict how peptide presentation changes under a range of genetic or physiological perturbations to the antigen presentation machinery.

## Author Contributions

DB conducted experimental work and RE conducted modeling work. AP, PC, and ND advised on modeling, and TE advised on experiments. The study was designed by TE, DB, ND, and AP. All authors wrote the manuscript.

## Conflict of Interest Statement

The authors declare that the research was conducted in the absence of any commercial or financial relationships that could be construed as a potential conflict of interest.

## Footnotes

^1^http://research.microsoft.com/gec.

^2^https://github.com/predictionmachines/Filzbach.
