# Supplementary figures and images for: A Mechanistic Model for Predicting Cell Surface Presentation of Competing Peptides by MHC Class I Molecules
Source: Front Immunol. 2018 Jul 5;9:1538. doi: 10.3389/fimmu.2018.01538 (PMC6041393; doi:10.3389/fimmu.2018.01538)

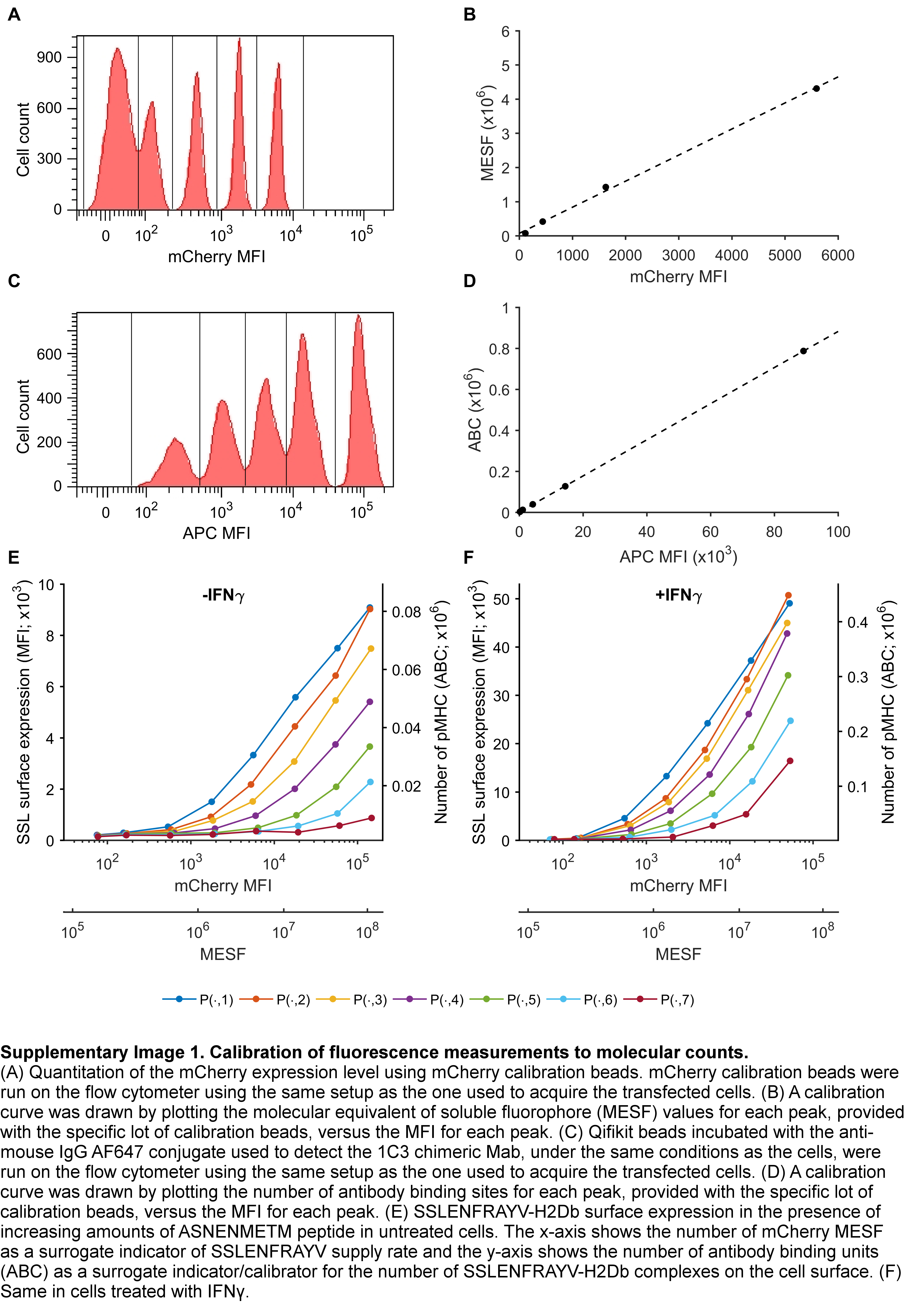

Supplement: Supplementary file 1 [file image_1.tif]

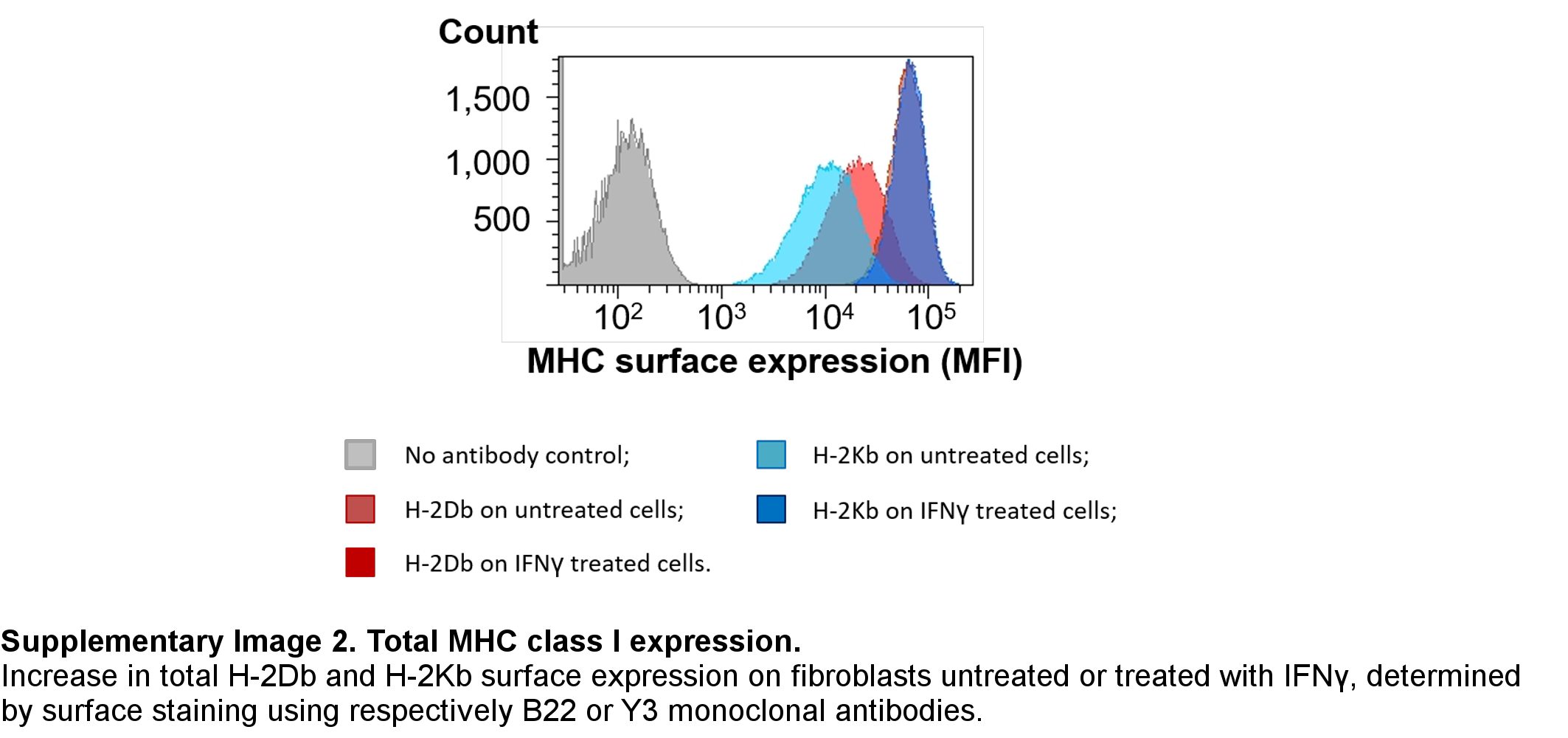

Supplement: Supplementary file 2 [file image_2.tif]

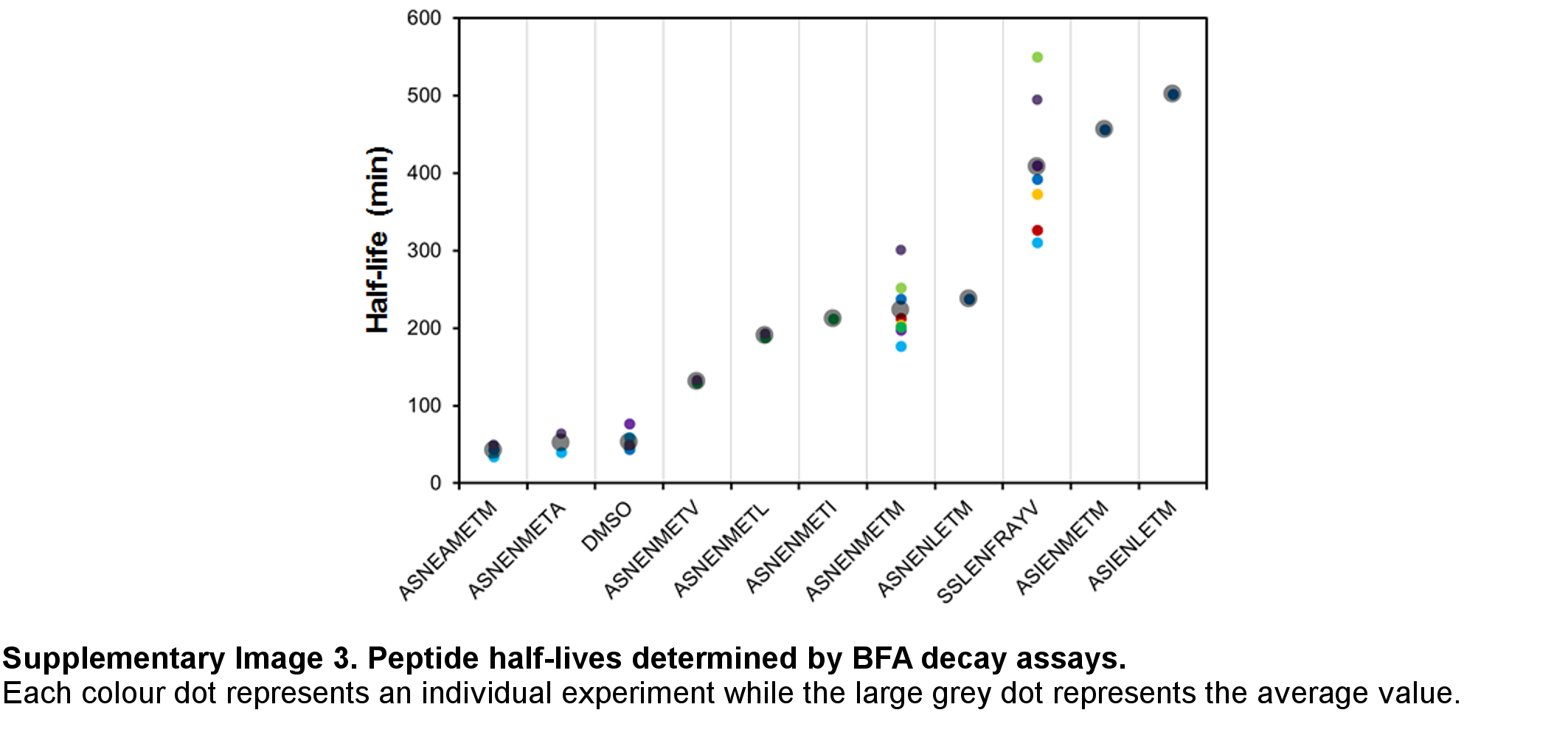

Supplement: Supplementary file 3 [file image_3.tif]

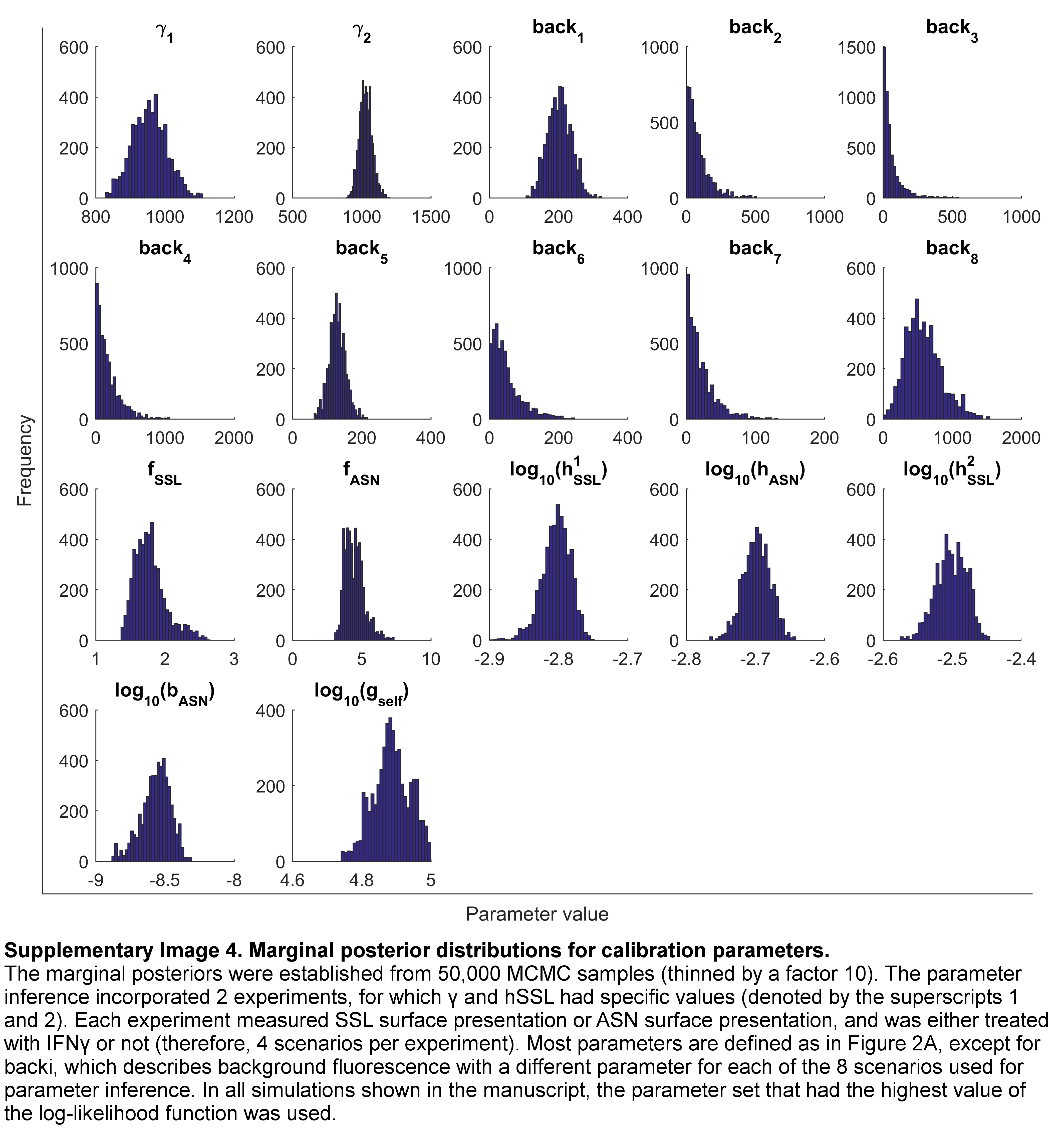

Supplement: Supplementary file 4 [file image_4.tif]

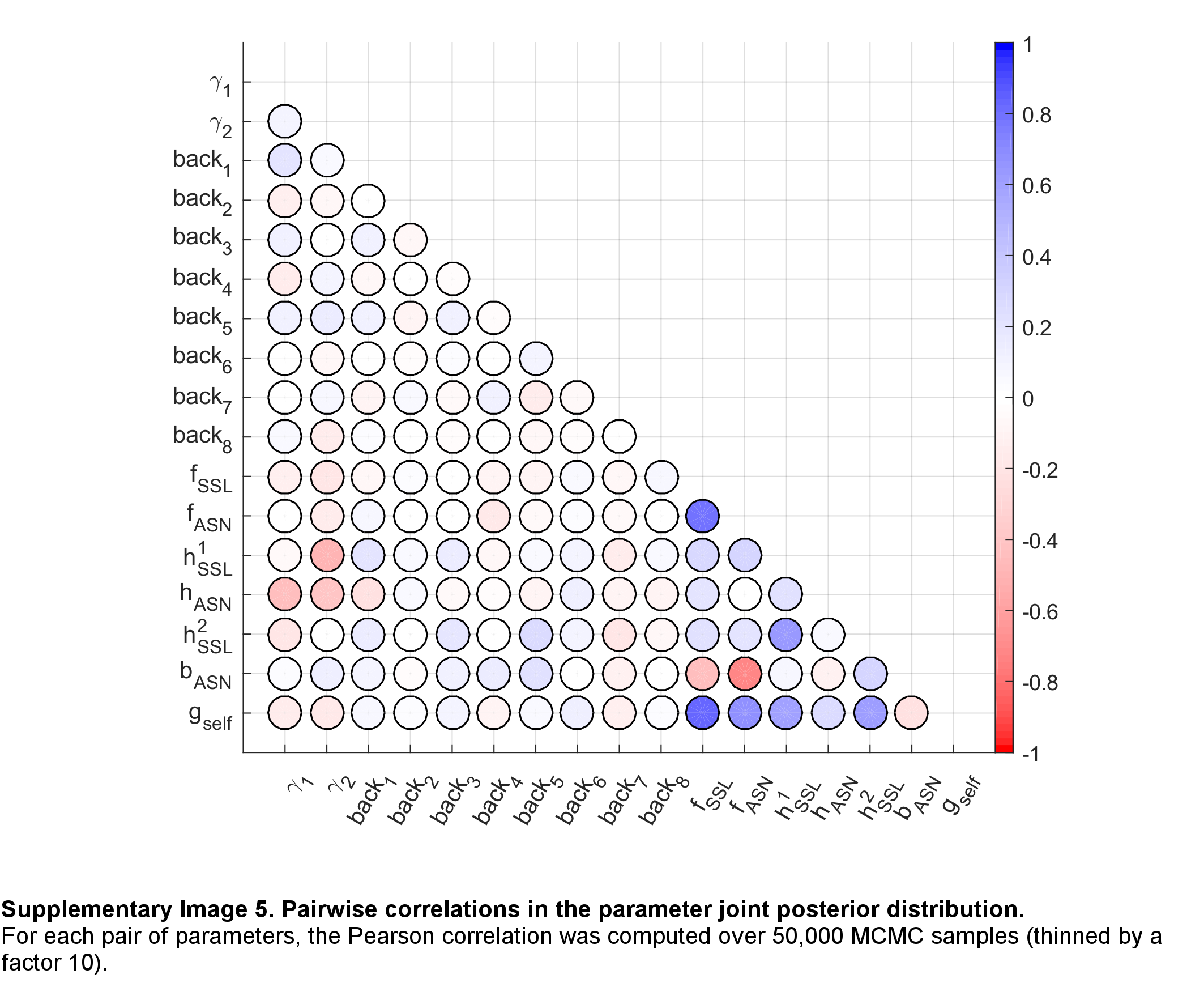

Supplement: Supplementary file 5 [file image_5.tif]

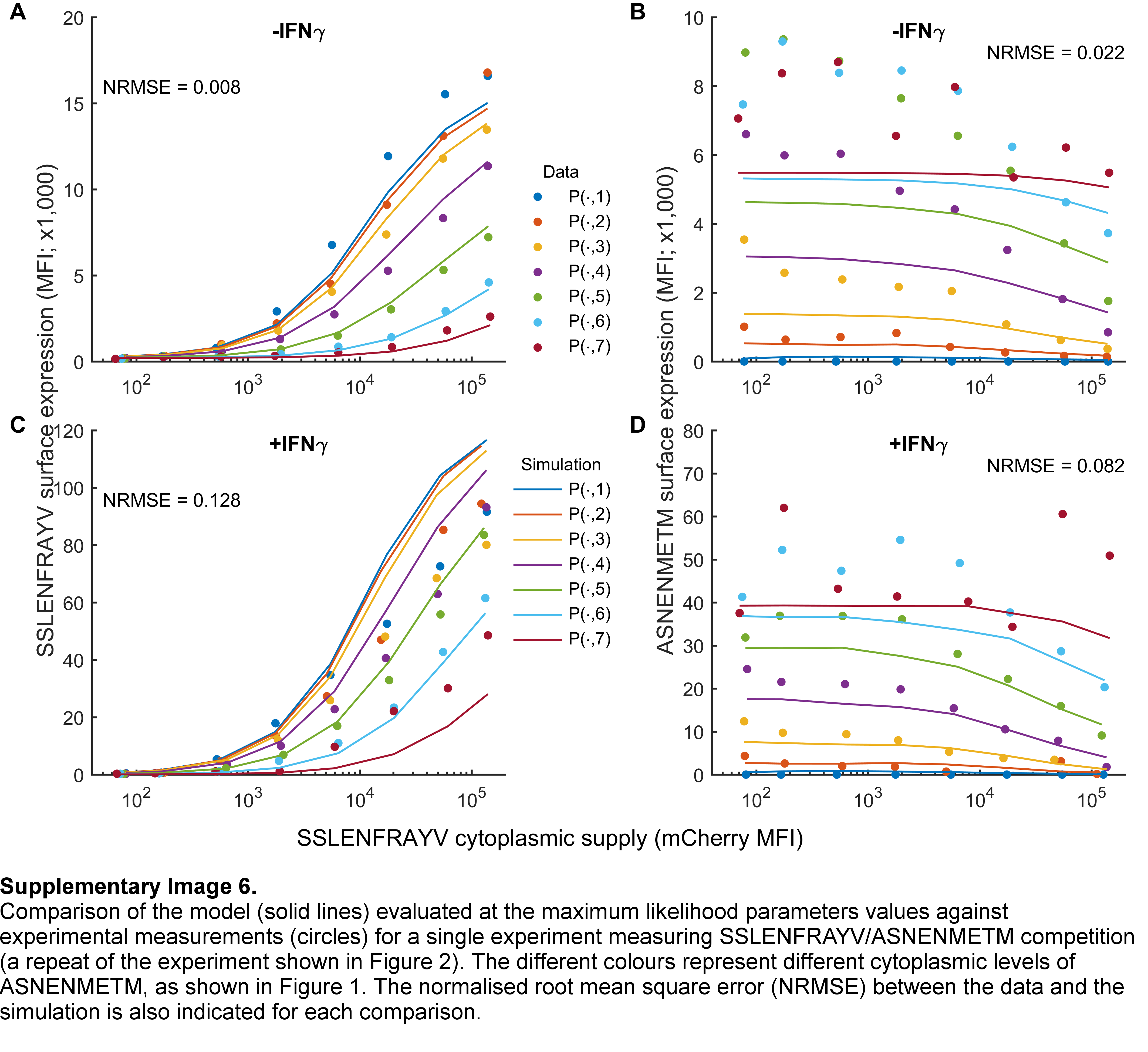

Supplement: Supplementary file 6 [file image_6.tif]

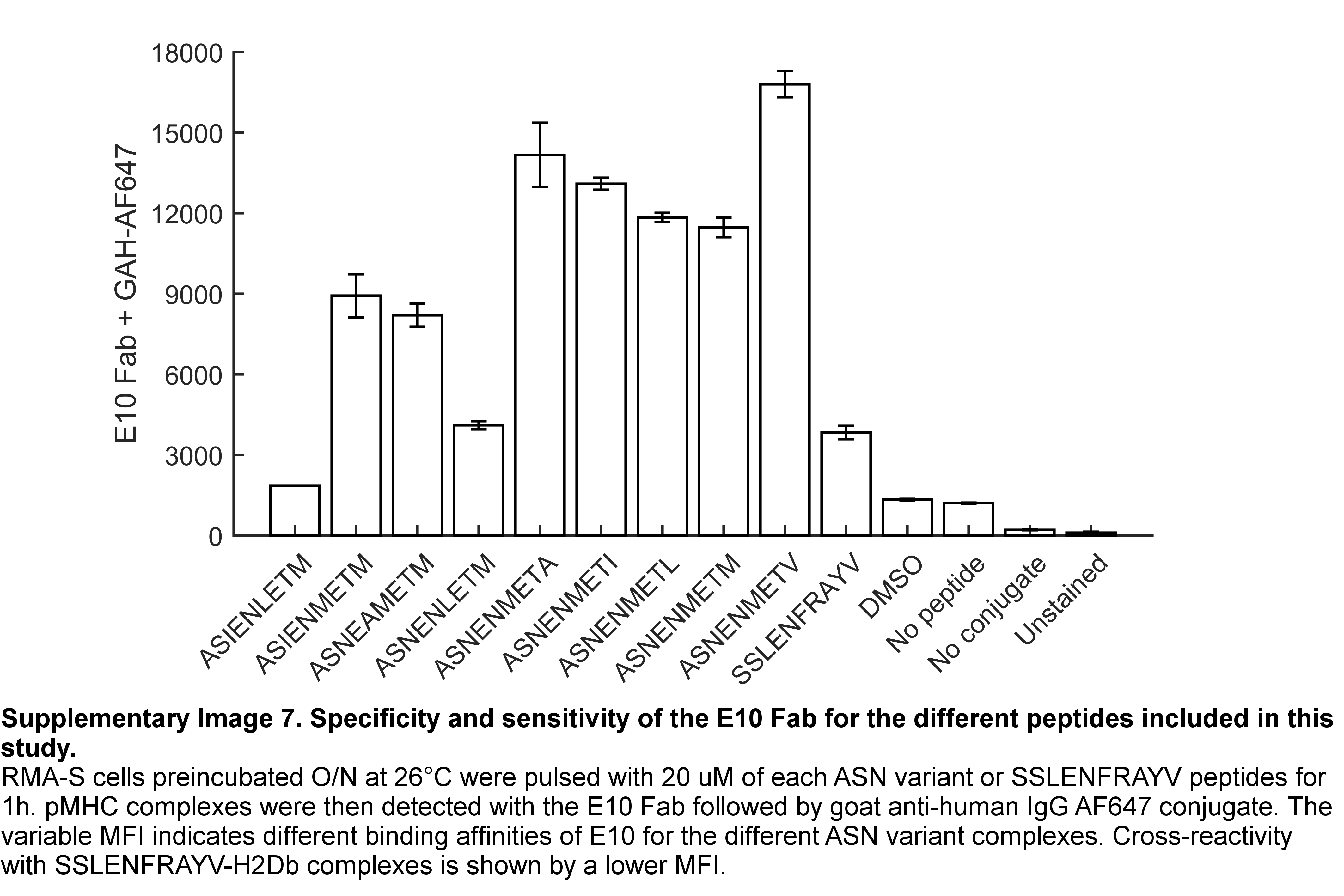

Supplement: Supplementary file 7 [file image_7.tif]

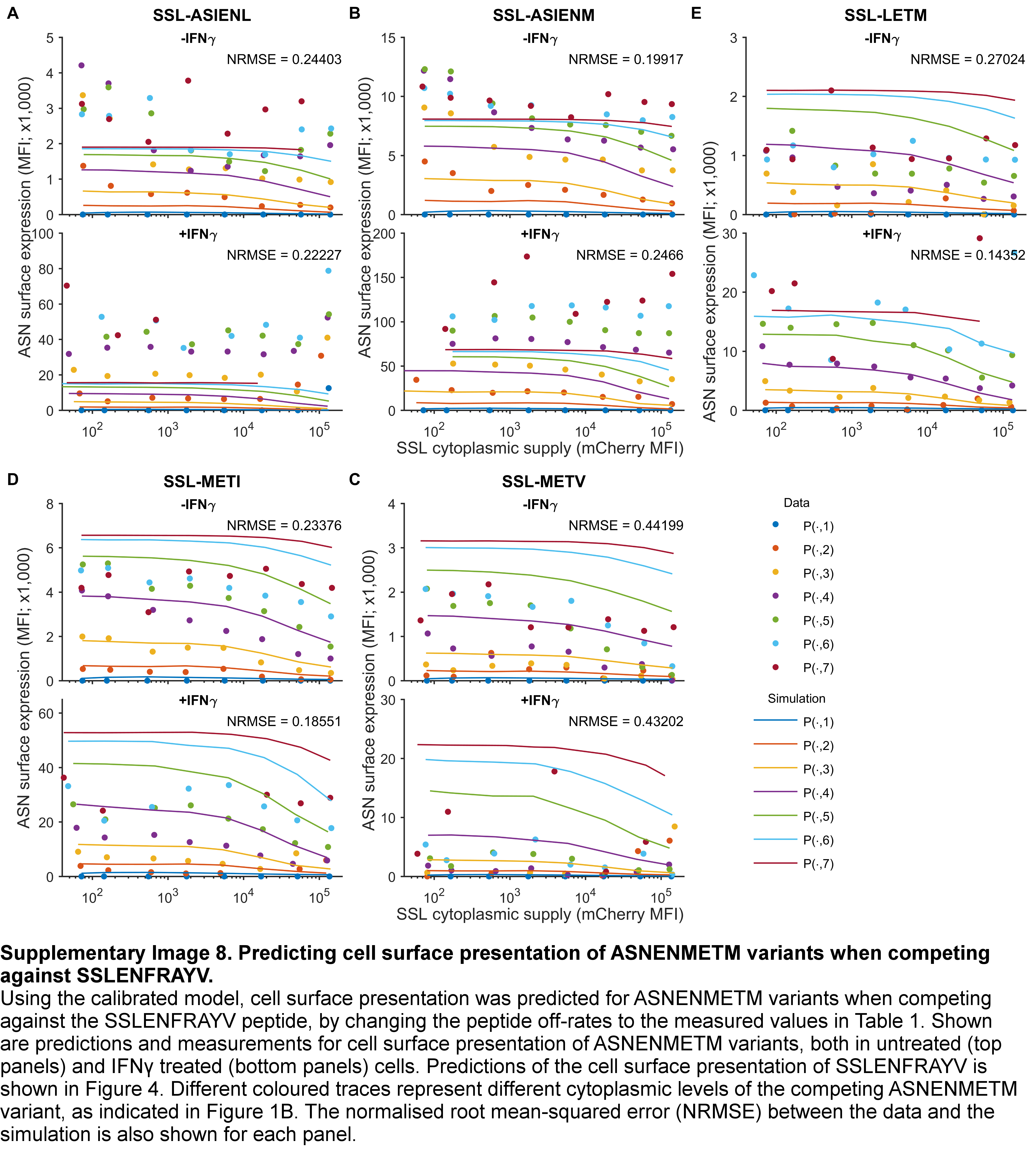

Supplement: Supplementary file 8 [file image_8.tif]

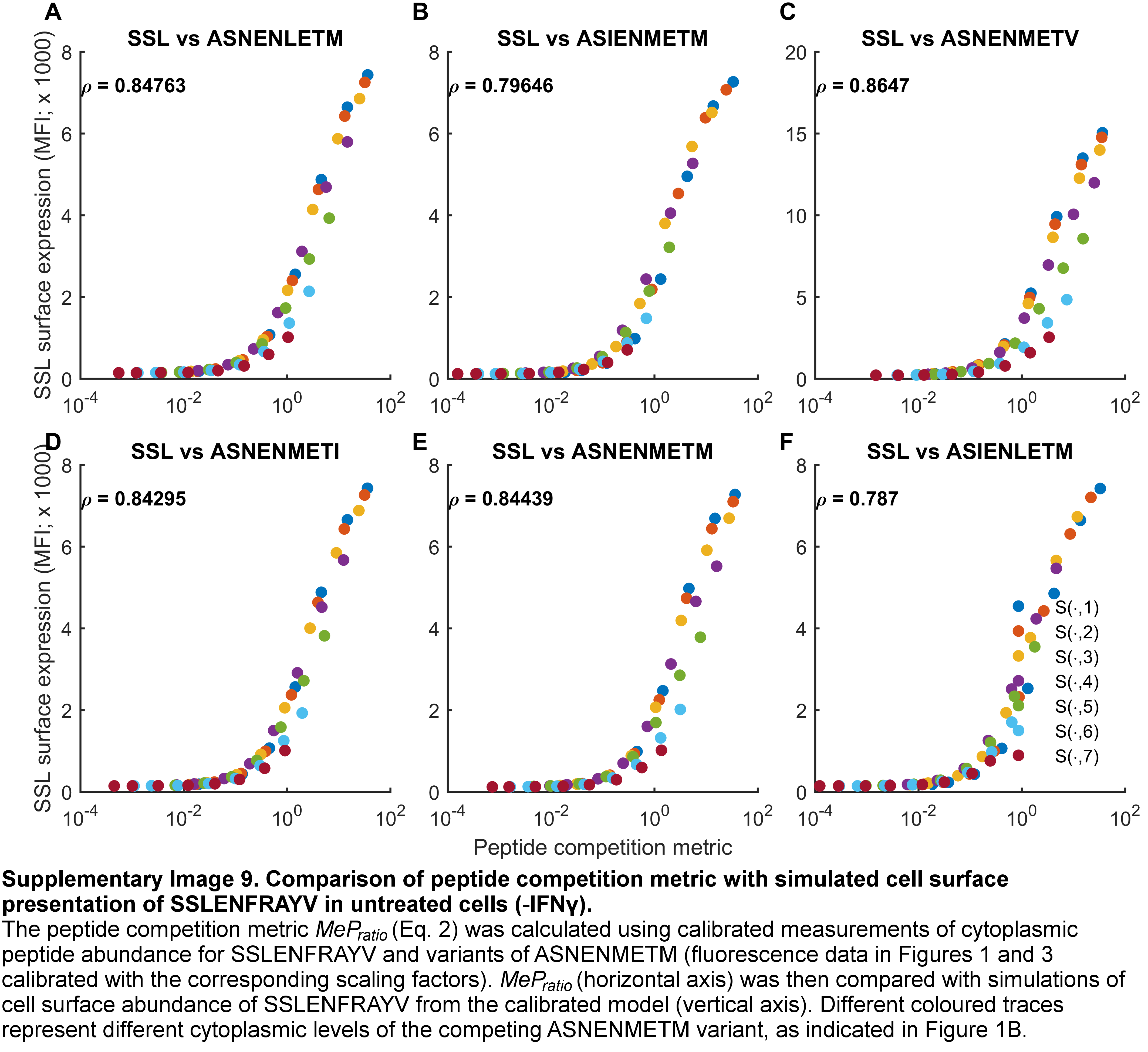

Supplement: Supplementary file 9 [file image_9.tif]

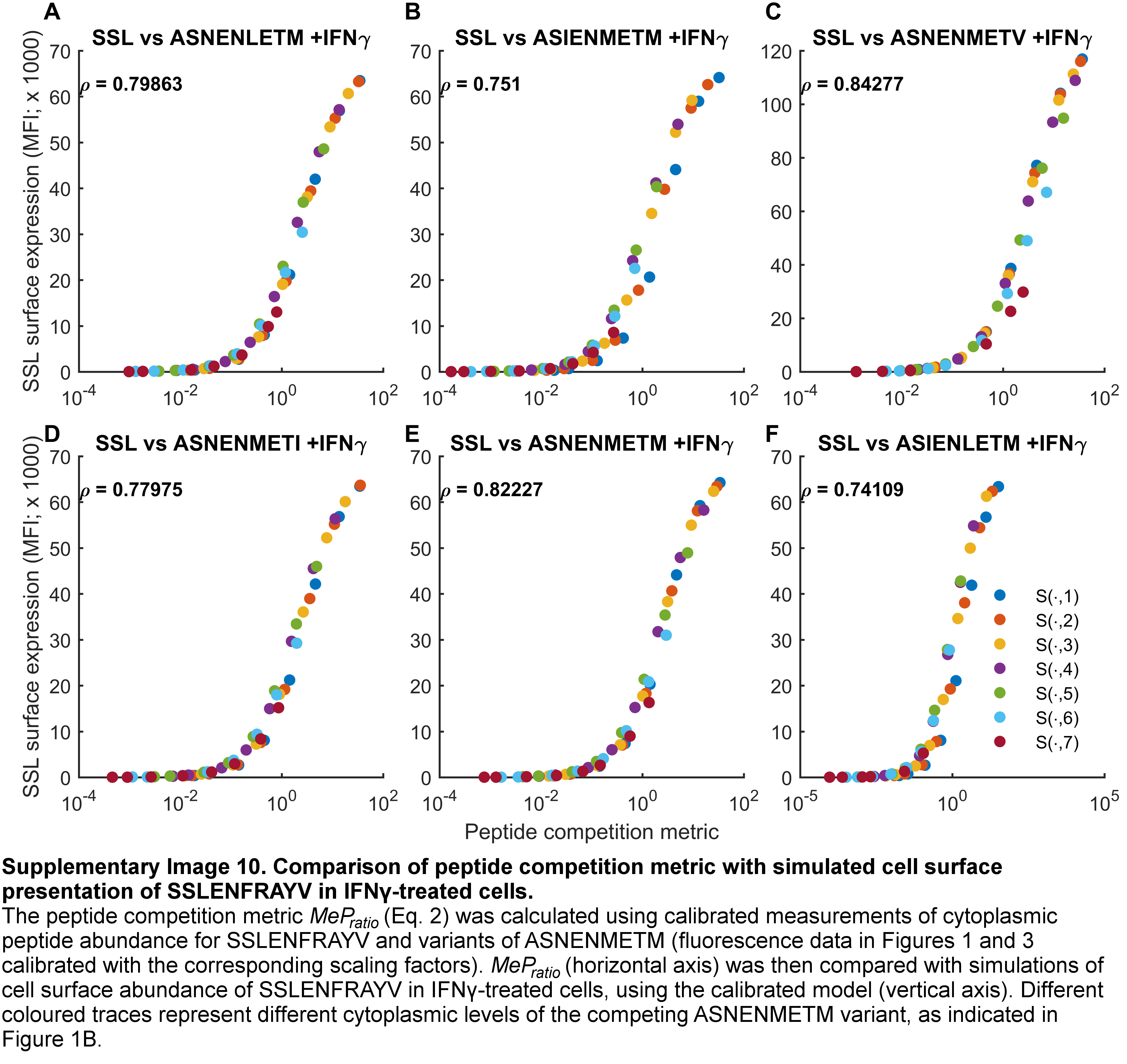

Supplement: Supplementary file 10 [file image_10.tif]

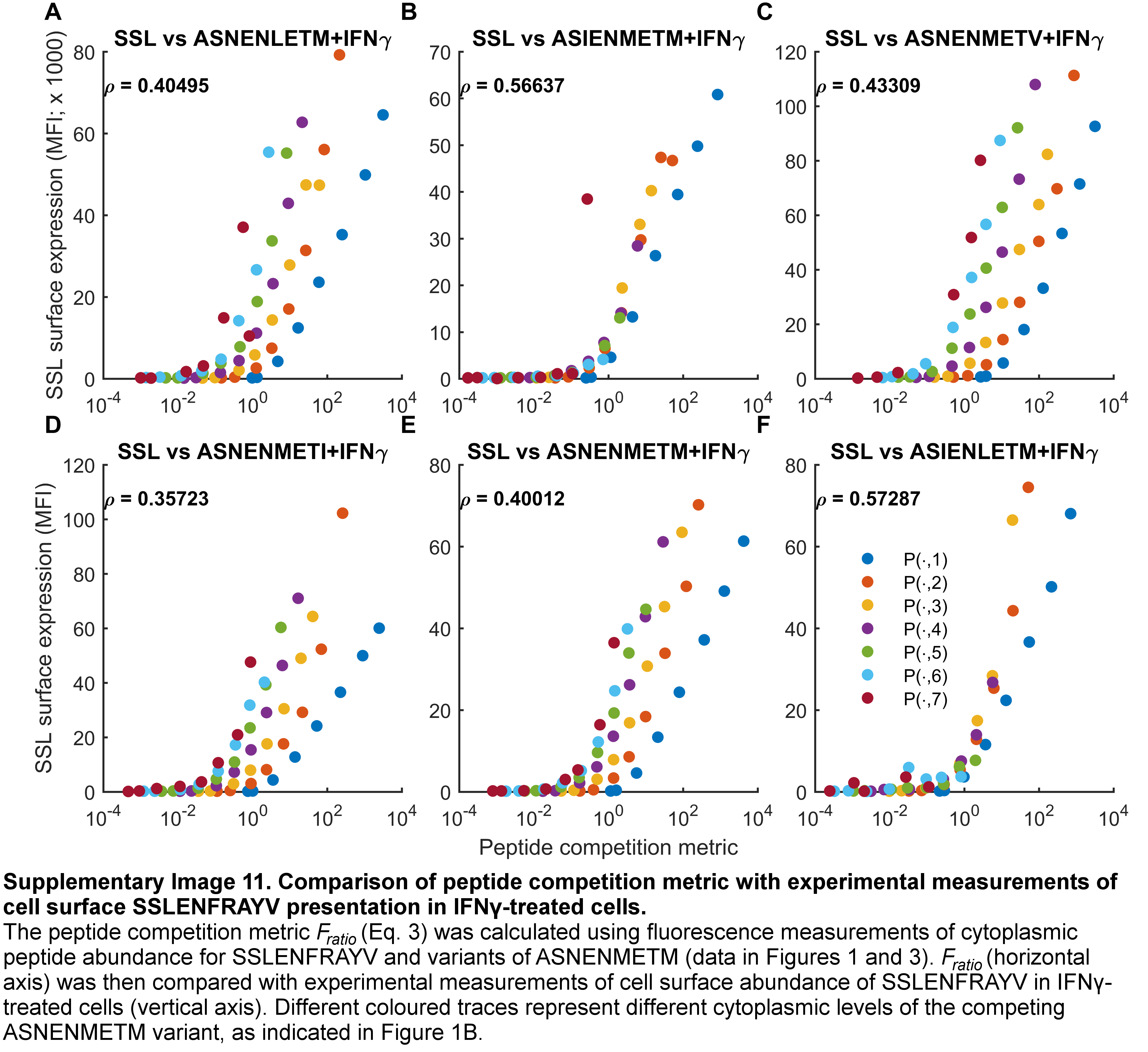

Supplement: Supplementary file 11 [file image_11.tif]
